# A single-cell atlas of the human respiratory system identifies a monocyte-centered HSPA1B^+^ inflammatory primed state associated with WTAP

**DOI:** 10.3389/fcimb.2026.1950659

**Published:** 2026-10-27

**Authors:** Ying Wang, Lin Zhu, Yejin Zhu, Guoping Chen

**Affiliations:** Department of Respiratory and Critical Care Medicine, Binhai County People’s Hospital, Binhai Clinical College, Yangzhou University Medical College, Yancheng, Jiangsu, China

**Keywords:** chronic obstructive pulmonary disease, HSPA1B, inflammation, monocyte, WTAP

## Abstract

Chronic obstructive pulmonary disease (COPD) is characterized by persistent pulmonary inflammation and tissue remodeling, yet the cellular states and regulatory mechanisms underlying monocyte functional heterogeneity remain incompletely understood. Here, we generate a high-resolution single-cell transcriptomic atlas of the human respiratory system and identify a diverse monocyte functional landscape in healthy lungs containing a HSPA1B^+^ inflammatory monocytes state. This population exhibits enhanced inflammatory programs, cytokine and chemokine activity, and shows a distinct position within the inferred transcriptional landscape of the continuous monocyte functional spectrum. Cell-cell communication analysis further reveals that HSPA1B^+^ inflammatory monocytes function as active communication nodes within a stromal–immune–vascular network, integrating MIF, extracellular matrix, and immune-regulatory signals while mediating CXCL and adhesion-associated interactions with surrounding cellular compartments. Comparative analysis of COPD lung tissues and bronchoalveolar lavage fluid demonstrates selective enrichment of this inflammatory monocyte program in disease-associated environments, whereas peripheral blood monocytes from healthy individuals and COPD patients show limited representation of this state. Integrated transcriptomic and m^6^A regulator-associated analyses identify WTAP, a core component of the m^6^A methyltransferase complex, as a factor associated with the inflammatory monocytes program. WTAP silencing in inflammatory monocyte models suppresses pro-inflammatory mediators while enhancing interferon-response and antigen-presentation programs. Collectively, these findings define an inflammatory monocyte state landscape in the human respiratory system and identify a WTAP-associated transcriptional program linked to monocyte inflammatory activation and functional remodeling in COPD. This study provides new insights into the organization of pulmonary immune states and the potential regulatory mechanisms underlying chronic inflammatory regulation in COPD.

## Introduction

Chronic obstructive pulmonary disease (COPD) is a major global health burden, affecting approximately 384 million individuals worldwide and representing the third leading cause of death globally (Adeloye et al., 2022; Wang et al., 2018; Zhang et al., 2024). Characterized by persistent airflow limitation, chronic respiratory symptoms, and progressive structural remodeling of the lung, COPD results from complex interactions between genetic susceptibility and environmental exposures, particularly cigarette smoke and air pollution (Barnes, 2019). Despite substantial advances in pharmacological interventions, including bronchodilators and anti-inflammatory therapies, current treatments remain largely focused on symptom management and functional improvement rather than disease modification (Celli and Wedzicha, 2019; Xiong and Guo, 2019). The incomplete therapeutic efficacy reflects a limited understanding of the cellular and molecular mechanisms that sustain chronic pulmonary inflammation and drive irreversible lung damage.

Chronic inflammation represents a central pathological feature of COPD and contributes to airway remodeling, alveolar destruction, and progressive decline in pulmonary function (Pappas et al., 2013). This inflammatory process involves complex interactions among immune and structural cell populations, including neutrophils, macrophages, lymphocytes, epithelial cells, and endothelial cells (Lan et al., 2025). Among these populations, monocytes and their differentiated macrophage progeny have emerged as critical regulators of pulmonary immune homeostasis and inflammatory remodeling (Ryan et al., 2023; Sui et al., 2025). Traditionally, monocyte biology has been interpreted according to classical and non-classical subsets, representing inflammatory recruitment and patrolling functions, respectively. However, increasing evidence indicates that monocytes exhibit substantial transcriptional and functional plasticity, occupying continuous immune states shaped by tissue environments rather than existing as rigidly defined populations. Recent single-cell transcriptomic studies have revealed diverse monocyte and macrophage states associated with inflammatory lung diseases, yet the functional organization of monocytes within the healthy human respiratory system and their contribution to chronic inflammatory disease progression remain incompletely understood.

The emergence of single-cell RNA sequencing (scRNA-seq) technologies has revolutionized the characterization of cellular heterogeneity within complex tissues and enabled high-resolution mapping of immune states in the human respiratory system (Deprez et al., 2020; Papalexi and Satija, 2018; Sikkema et al., 2023; Travaglini et al., 2020; Xu et al., 2022). Large-scale lung cell atlases, including the Human Lung Cell Atlas (HLCA), have provided comprehensive references of epithelial, stromal, and immune cell populations across healthy and diseased conditions, including COPD, COVID-19, and lung cancer (Sikkema et al., 2023). These studies have identified disease-associated macrophage and monocyte populations and highlighted extensive immune remodeling during pulmonary disease progression (Huang et al., 2022; Sin, 2024). However, current studies have largely focused on inflammatory states observed in diseased tissues, whereas the physiological organization of monocyte functional states in healthy lungs remains poorly defined. In particular, whether inflammatory monocyte programs are exclusively induced during pathological conditions or represent pre-existing immune states embedded within healthy tissue to maintain immune surveillance and rapid environmental responses remains unknown.

Beyond cellular heterogeneity, increasing evidence suggests that epigenetic and epitranscriptomic mechanisms play fundamental roles in regulating immune cell plasticity and inflammatory adaptation (Pinello et al., 2024; Srivastava et al., 2025; Zhu et al., 2023). N6-methyladenosine (m^6^A) RNA modification, the most abundant internal modification in mammalian mRNA, has emerged as an important regulatory layer controlling RNA stability, translation efficiency, and cellular responses. m^6^A modification is dynamically regulated by methyltransferase “writer” complexes, including METTL3, METTL14, and WTAP, demethylase “eraser” enzymes such as FTO and ALKBH5, and RNA-binding “reader” proteins including YTHDF and YTHDC family members (Zaccara et al., 2019). Recent studies have demonstrated that m^6^A signaling regulates diverse immune processes, including innate immune activation, inflammatory cytokine production, and immune cell differentiation (Karandashov et al., 2024). However, how m^6^A regulatory networks shape monocyte functional states within tissue-specific inflammatory environments, particularly during chronic respiratory inflammation, remains largely unexplored. Among m^6^A regulatory components, WTAP (Wilms tumor 1-associated protein) represents an essential regulatory component of the m^6^A methyltransferase complex and functions as a scaffold facilitating METTL3-METTL14 complex assembly and RNA methylation regulation. Beyond its canonical role in m^6^A deposition, emerging evidence suggests that WTAP may actively participate in immune regulation by controlling inflammatory transcriptional programs and cellular state transitions (Ping et al., 2014). Increased WTAP expression has been reported in several chronic inflammatory disorders, including rheumatoid arthritis, atherosclerosis, and inflammatory bowel disease, where it is associated with enhanced inflammatory responses and immune activation (Luo et al., 2025; Yan and Wan, 2025). These findings suggest that WTAP may represent a molecular link between epitranscriptomic regulation and inflammatory immune states. However, whether WTAP-associated m^6^A programs regulate human lung monocyte functional states and contribute to COPD-associated inflammatory remodeling remains unknown.

Here, we analyzed single-cell transcriptomic datasets from multiple anatomical regions of the healthy human respiratory system, including lung parenchyma, respiratory airways, and nasal tissues, with COPD patient lung scRNA-seq datasets to define the cellular and molecular architecture of respiratory monocyte states. Through high-resolution monocyte profiling, functional state characterization, trajectory analysis, cell–cell communication mapping, and m^6^A regulator-associated analysis, we identify HSPA1B^+^ inflammatory monocytes as an inflammatory monocyte state observed within the healthy respiratory system. This population exhibits classical monocyte characteristics, enhanced inflammatory programs, and distinct signaling patterns, and occupies a distinct functional position along the monocyte state continuum. Importantly, we demonstrate that this inflammatory monocyte state is more highly represented in COPD lungs, suggesting that chronic pulmonary inflammation may involve increased representation and remodeling of inflammatory immune programs rather than generation of entirely novel inflammatory populations. Furthermore, we identify a WTAP-associated transcriptional program linked to inflammatory monocyte activation, providing insights into the potential role of WTAP-associated regulation in monocyte functional plasticity and chronic respiratory inflammation. Collectively, our findings establish a continuous monocyte state landscape in the human respiratory system and reveal WTAP-associated transcriptional regulation as a potential molecular framework associated with inflammatory immune remodeling in COPD.

## Materials and methods

### Data collection and processing of human respiratory single-cell transcriptomic datasets

Publicly available human respiratory single-cell RNA sequencing (scRNA-seq) datasets were collected from the CELLxGENE Discover platform and the Gene Expression Omnibus (GEO) database to establish a reference atlas of human respiratory cellular states and investigate COPD-associated monocyte alterations. The healthy respiratory reference atlas was constructed using the integrated cell atlas of the human lung in health and disease (core dataset) from CELLxGENE, which includes normal respiratory tissues from multiple anatomical compartments, including lung parenchyma, respiratory airway, and nasal tissues. For COPD-associated analyses, lung tissue scRNA-seq data from COPD patients and controls were obtained from GSE173896 and the Shaykhiev dataset (Rustam et al., 2023) to evaluate disease-related alterations in pulmonary cellular composition and monocyte functional states. In addition, GSE249584, consisting of peripheral blood mononuclear cell (PBMC) scRNA-seq profiles from COPD patients and healthy individuals, was analyzed to assess whether identified inflammatory monocyte programs were detectable in circulating immune compartments. For the BALF analysis, we used a primary human respiratory scRNA-seq dataset generated in the Shaykhiev laboratory (Weill Cornell Medical College) from NLD and COPD subjects (Rustam et al., 2023). Samples were collected from three independent tissue procurement sites and included freshly prepared single-cell suspensions from proximal and distal airways and terminal bronchoalveolar units.

Raw expression matrices and corresponding metadata were obtained from public repositories. All datasets were processed using a standardized workflow, including quality control, normalization, dimensionality reduction, batch correction, clustering, and cell-type annotation. Cells with fewer than 200 detected genes or more than 20% mitochondrial gene expression were excluded. Clustering was performed using the Louvain algorithm with a resolution of 0.5. Integrated datasets were subsequently used for characterization of respiratory cellular heterogeneity, monocyte functional states, trajectory inference, cell-cell communication analysis, and evaluation of disease-associated inflammatory remodeling.

### Single-cell RNA sequencing data processing and cell-type annotation

Single-cell transcriptomic data were processed using R software (version 4.2.1) with the Seurat package (version 4.3.0). Cells with low-quality transcriptomic profiles were excluded according to predefined quality control criteria. Specifically, cells with excessive mitochondrial gene expression (>20%), low transcript counts, or abnormal gene detection numbers were removed before downstream analysis.

Following quality filtering, gene expression matrices were normalized using the NormalizeData function in Seurat. Highly variable genes were identified using the variance-stabilizing transformation method implemented in FindVariableFeatures. The normalized expression matrix was subsequently scaled using ScaleData, and principal component analysis (PCA) was performed for dimensionality reduction.

To integrate multiple respiratory datasets generated from different individuals and studies, batch correction was performed using Harmony based on PCA embeddings. The corrected embeddings were used for Uniform Manifold Approximation and Projection (UMAP) visualization. Cell clusters were identified by constructing a shared nearest-neighbor graph using FindNeighbors, followed by graph-based clustering using FindClusters.

Cell identities were assigned through a combination of canonical marker gene expression analysis and reference-based annotation. Major respiratory cell populations, including epithelial cells, endothelial cells, fibroblasts, macrophages, monocytes, dendritic cells, T/NK cells, B/plasma cells, and other immune populations, were annotated based on established lineage-specific markers. All subsequent analyses were performed using the annotated and batch-corrected datasets.

### Identification and characterization of monocyte subpopulations

To investigate monocyte heterogeneity within the human respiratory system, monocytes were extracted from the integrated dataset and subjected to independent high-resolution reclustering analysis. Dimensionality reduction and clustering were repeated specifically within the monocyte compartment. Distinct monocyte subpopulations were identified based on transcriptomic signatures and expression patterns of established classical and non-classical monocyte markers, including CD14 and FCGR3A.

Functional characteristics of each monocyte population were evaluated using predefined gene signatures representing classical monocytes, non-classical monocytes, inflammatory activation, interferon response, and antigen presentation programs. Signature scores were calculated using the AddModuleScore function implemented in Seurat.

Inflammatory activity was quantified based on inflammatory gene modules, and subpopulations exhibiting distinct inflammatory characteristics were further investigated. The HSPA1B^+^ inflammatory monocyte population was identified based on differential expression analysis and characteristic enrichment of inflammatory transcriptional programs.

### Differential expression analysis and functional enrichment analysis

Differentially expressed genes (DEGs) between cell populations or experimental groups were identified using the FindMarkers or FindAllMarkers functions in Seurat based on the Wilcoxon rank-sum test. Genes were considered significantly differentially expressed when they satisfied the following criteria: adjusted *P* value <0.05 and absolute log2 fold change >0.25 unless otherwise specified. Functional enrichment analysis of DEG lists was performed using the clusterProfiler R package. Gene Ontology (GO) enrichment analysis was conducted across Biological Process (BP), Cellular Component (CC), and Molecular Function (MF) categories. Significantly enriched pathways were identified based on adjusted *P* values <0.05.

### Monocyte trajectory analysis

To investigate potential functional transitions among monocyte states, pseudotime trajectory analysis was performed using Monocle2. Highly variable genes associated with monocyte state variation were selected for trajectory construction. Cells were ordered along pseudotime according to transcriptional similarity, allowing reconstruction of potential developmental and activation trajectories among monocyte subpopulations. Genes dynamically changing along pseudotime were identified and subjected to GO enrichment analysis to characterize stage-specific biological programs associated with monocyte state transitions.

### Cell–cell communication analysis

Intercellular communication networks within healthy respiratory tissues were inferred using the CellChat R package based on ligand–receptor interaction analysis. All annotated cell populations, including epithelial cells, endothelial cells, fibroblasts, immune populations, macrophages, and monocyte subpopulations, were incorporated into the communication analysis. CellChat was used to estimate interaction probabilities and communication strengths based on curated ligand–receptor databases. Global communication patterns were evaluated by comparing interaction numbers, signaling strength, and information flow among cell populations. Pathway-level communication activity was assessed to identify dominant signaling programs contributing to respiratory tissue homeostasis. Incoming and outgoing signaling analyses were performed to define major signal-receiving and signal-producing populations. Specific ligand–receptor interactions involving HSPA1B^+^ inflammatory monocytes were further examined to identify communication axes associated with immune regulation, chemokine signaling, extracellular matrix sensing, and inflammatory activation.

### m^6^A regulatory network analysis

To investigate the potential involvement of RNA methylation regulation in monocyte functional states, a curated panel of m^6^A-related regulatory genes was analyzed. The analyzed gene set included m^6^A methyltransferase components (*METTL3*, *METTL14*, *WTAP*, *RBM15*, *RBM15B*, and *ZC3H13*), demethylases (*FTO* and *ALKBH5*), and m^6^A reader proteins (*YTHDF1*, *YTHDF2*, *YTHDF3*, *YTHDC1*, *YTHDC2*, *IGF2BP1*, *IGF2BP2*, *IGF2BP3*, *HNRNPC*, *HNRNPA2B1*, *FMR1*, *LRPPRC*, *ELAVL1*, and *CBLL1*). Expression patterns of m^6^A regulators across monocyte subpopulations were evaluated, and unsupervised clustering based on m^6^A regulatory gene expression was performed to determine whether m^6^A-related signatures could distinguish functional monocyte states.

### WTAP-associated functional state analysis

Monocytes were stratified into WTAP-high and WTAP-low populations according to WTAP expression levels. Differential expression analysis was performed between these two groups using the Wilcoxon rank-sum test. Differential genes were subsequently analyzed using GO enrichment analysis to identify biological programs associated with WTAP expression. The relationships between m^6^A regulators and monocyte functional states were assessed using Spearman correlation analysis. Correlations between regulator expression levels and inflammatory, interferon-response, and antigen-presentation signatures were calculated. Pathway activity scores were generated using AddModuleScore, and differences between WTAP-high and WTAP-low populations were statistically evaluated.

### THP-1 monocyte inflammatory activation and WTAP silencing

Human THP-1 monocytes (ATCC) were cultured in RPMI 1640 medium supplemented with 10% FBS. For WTAP loss-of-function experiments, THP-1 cells were first transfected with WTAP-specific siRNA or non-targeting control siRNA using Lipofectamine™ 2000. After 24 h of transfection, cells were stimulated with lipopolysaccharide (LPS, 100 ng/mL) and interferon-γ (IFN-γ, 20 ng/mL) to induce an inflammatory monocyte-like state. Following 24 h of stimulation, cells were collected for RNA extraction and downstream transcriptional analysis. The WTAP-specific siRNA used in this study was synthesized by RiboBio (Guangzhou, Guangdong, China), with the sequence 5’-AAUGAAAGGUGAACUGGAA-3’, as previously reported (Han et al., 2024). The experiments were independently repeated three times.

WTAP depletion was evaluated at both the transcript and protein levels. For intracellular WTAP staining, Cells were fixed and permeabilized before incubation with an anti-WTAP antibody (ab195380; Abcam). WTAP staining was subsequently detected using Goat Anti-Rabbit IgG Alexa Fluor™ 594 (A-11037; Thermo Fisher Scientific) as the secondary antibody, following the manufacturer’s recommended protocol. Protein-level WTAP expression was assessed by flow cytometry, and cell viability was evaluated using a viability dye-based assay. The proportion of WTAP-positive cells and cell viability were quantified following the indicated treatments.

### Primary human monocyte isolation and WTAP silencing

Peripheral blood mononuclear cells (PBMCs) were commercially obtained from Beijing Mumi Xing Biotechnology Co., Ltd. (Leuko Pak, Cat. No. PB007-2) and were not obtained through direct donor recruitment or blood collection by the authors. For the present study, PBMCs were used for isolation of primary human monocytes. Primary human monocytes were isolated from the PBMC preparation using the EasySep™ Human Monocyte Isolation Kit (STEMCELL Technologies, Vancouver, Canada; Cat. No. 19359) according to the manufacturer’s instructions. Briefly, PBMCs were resuspended in EasySep™ Buffer at a concentration of 5 × 10^7^ cells/mL. The EasySep™ Human Monocyte Isolation Cocktail and EasySep™ Human Platelet Removal Cocktail were added at 50 μL/mL of cell suspension, followed by incubation at room temperature for 5 min. EasySep™ D Magnetic Particles for Human Monocytes were then added at 50 μL/mL, and the cell suspension was incubated at room temperature for an additional 5 min. The labeled cell suspension was subsequently placed in an EasySep™ Magnet for 2.5 min, and the enriched monocyte fraction was collected into a new tube. The isolated cells were immediately used for subsequent experiments.

For WTAP loss-of-function experiments, isolated primary human monocytes were transfected with WTAP-specific siRNA or non-targeting control siRNA using the same transfection strategy described for THP-1 cells. Following siRNA transfection, cells were stimulated with lipopolysaccharide (LPS, 100 ng/mL) and interferon-γ (IFN-γ, 20 ng/mL) to induce an inflammatory activation state. Following the indicated treatments, cells were collected for RNA extraction and quantitative real-time PCR (qRT-PCR) analysis.

### Quantitative real-time PCR

Total RNA was extracted from cultured cells using TRIzol reagent (Invitrogen) following the standard protocol. Equal amounts of RNA were reverse-transcribed into cDNA using a commercial cDNA synthesis system (Takara Bio). Quantitative real-time PCR was performed using TB Green-based SYBR Green chemistry (Takara Bio) on a QuantStudio real-time PCR system (Applied Biosystems). Relative transcript abundance was calculated using the comparative threshold cycle method (2^-ΔΔCt^) and normalized to the expression of β-ACTIN. Primer sequences are provided in **Table 1**.

**Table 1:** Primer sequences used for quantitative real-time PCR analysis, related to Materials and methods.

Primer sequence.		
Name	Forward (5’ to 3’)	Reverse (5’ to 3’)
*β-ACTIN*	CACCATTGGCAATGAGCGGTTC	AGGTCTTTGCGGATGTCCACGT
*WTAP*	GCAACAACAGCAGGAGTCTGCA	CTGCTGGACTTGCTTGAGGTAC
*TNF*	CTCTTCTGCCTGCTGCACTTTG	ATGGGCTACAGGCTTGTCACTC
*IL1B*	CCACAGACCTTCCAGGAGAATG	GTGCAGTTCAGTGATCGTACAGG
*CXCL8*	GAGAGTGATTGAGAGTGGACCAC	CACAACCCTCTGCACCCAGTTT
*IL6*	AAGAAGTAGGCTGGCACGACGT	GCGGATGTTCTCCACAATGGAC
*IFIT3*	CCTGGAATGCTTACGGCAAGCT	GAGCATCTGAGAGTCTGCCCAA
*RSAD2*	CCAGTGCAACTACAAATGCGGC	CGGTCTTGAAGAAATGGCTCTCC
*HL1-DRA*	AGCTGTGGACAAAGCCAACCTG	CTCTCAGTTCCACAGGGCTGTT
*HLA-DRB1*	GAGCAAGATGCTGAGTGGAGTC	CTGTTGGCTGAAGTCCAGAGTG
*CD74*	AAGCCTGTGAGCAAGATGCGCA	AGCAGGTGCATCACATGGTCCT
*CIITA*	CTACTTCAGGCAGCAGAGGAGA	GCTGTGTCTTCCGAGGAACTTC
*HLA-DPA1*	ATCCAGCGTTCCAACCACACTC	CGTTGAGCACTGGTGGGAAGAA
*CTSS*	TGGATCACCACTGGCATCTCTG	GCTCCAGGTTGTGAAGCATCAC
*ISG15*	CTCTGAGCATCCTGGTGAGGAA	AAGGTCAGCCAGAACAGGTCGT
*MX1*	GGCTGTTTACCAGACTCCGACA	CACAAAGCCTGGCAGCTCTCTA
*IFIT1*	GCCTTGCTGAAGTGTGGAGGAA	ATCCAGGCGATAGGCAGAGATC
*OAS1A*	AGGAAAGGTGCTTCCGAGGTAG	GGACTGAGGAAGACAACCAGGT

### Statistical analysis

All statistical analyses were performed using R software (version 4.3.0) and GraphPad Prism (version 8.3.1; GraphPad Software). Statistical methods were selected according to the characteristics of each dataset and are specified in the corresponding figure legends. For comparisons between two independent groups, two-sided statistical tests were applied as appropriate. Continuous variables are presented as mean ± standard deviation (SD) unless otherwise indicated. The number of samples, biological replicates, and statistical approaches used for individual analyses are provided in the respective figure legends. A *P* value <0.05 was considered statistically significant. Significance levels were denoted as follows: n.s., not significant; *, *P* < 0.05; **, *P* < 0.01; ***, *P* < 0.001; ****, *P* < 0.0001.

## Results

### Single-cell transcriptomic profiling of the healthy human respiratory system reveals regional cellular heterogeneity and inflammatory preparedness of monocytes.

To systematically characterize the cellular composition and immune microenvironmental heterogeneity across distinct anatomical regions of the healthy human respiratory system, we analyzed single-cell RNA sequencing datasets from healthy donors covering the lung parenchyma, nasal mucosa, and respiratory airways, thereby generating a comprehensive single-cell transcriptomic atlas spanning multiple structural compartments of the respiratory tract (**Figure 1A**, **Table 2**). Through unsupervised clustering combined with canonical marker gene expression patterns, we identified nine major cell populations, including B/plasma cells, dendritic cells, endothelial cells, epithelial cells, fibroblasts, macrophages, monocytes, other immune cells, and T/NK cells (**Figure 1A**). Cell type-specific marker gene expression analysis further confirmed the robustness of cell annotation, with each cellular population displaying distinct molecular signatures characteristic of their respective lineages (**Figure 1B**). To evaluate the quality of dataset integration, we performed dimensionality reduction analysis based on sample origin. Cells from different donors and anatomical regions exhibited extensive mixing in the UMAP space without apparent donor- or tissue-specific clustering bias, indicating effective removal of technical variation and supporting the reliability of the integrated dataset for subsequent comparative analyses (**Figure 1C**).

**Figure 1:**
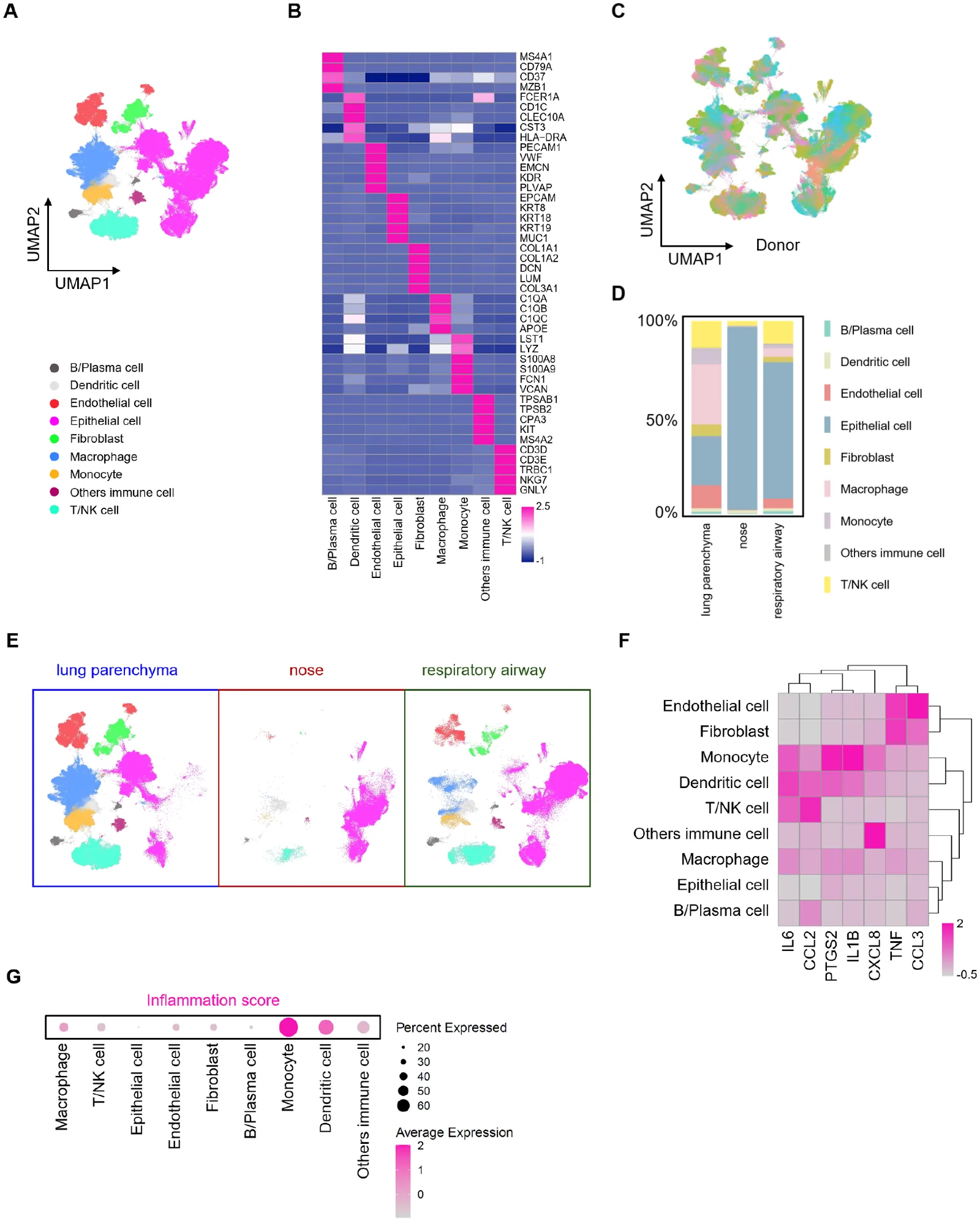
Single-cell atlas of the healthy human respiratory system reveals spatially organized cellular heterogeneity and monocyte-associated inflammatory priming during tissue homeostasis. **(A)** UMAP visualization of the integrated single-cell transcriptomic landscape of the healthy human respiratory system. Major cellular compartments were identified, including epithelial cells, endothelial cells, fibroblasts, macrophages, monocytes, dendritic cells, mast cells, neutrophils, T/NK cells, B/plasma cells, and others immune cell populations. **(B)** Heatmap showing the expression patterns of representative marker genes used for cell type annotation across identified cellular populations. **(C)** UMAP projection showing the distribution of cells from different donors, confirming successful dataset integration and effective mitigation of batch effects. **(D)** Relative abundance of major cell populations across distinct anatomical regions of the healthy human respiratory system. **(E)** UMAP representation highlighting the distribution of major cell populations across different respiratory tissue compartments. **(F)** Heatmap illustrating the expression patterns of representative inflammatory marker genes across major annotated cell populations. **(G)** DotPlot showing the inflammation score across major annotated cell populations.

**Table 2:** Summary of human respiratory single-cell RNA sequencing datasets analyzed in this study, related to Materials and methods.

Dataset source	Accession number	Dataset type	Sample population
CELLxGENE Discover	https://cellxgene.cziscience.com/e/688185ad-11c2-4172-a53a-f4f1f4076860/	scRNA-seq	Healthy human respiratory samples
Gene Expression Omnibus (GEO)	GSE173896	scRNA-seq	COPD patients and controls
Shaykhiev dataset	https://cellxgene.cziscience.com/collections/4f30b962-d49b-4624-a233-64f048cf8632	scRNA-seq	COPD patients and controls
Gene Expression Omnibus (GEO)	GSE249584	scRNA-seq	COPD patients and healthy individuals

We next investigated the cellular composition differences across distinct respiratory anatomical regions. Marked regional heterogeneity in cellular distribution was observed among the lung parenchyma, nasal mucosa, and respiratory airways (**Figure 1D**). The nasal mucosa and respiratory airways were predominantly composed of epithelial cells, consistent with their specialized role as frontline interfaces exposed to environmental stimuli and their dependence on epithelial barrier programs to maintain local tissue homeostasis. In contrast, the lung parenchyma exhibited a substantially more complex cellular landscape, with macrophages representing the dominant population, followed by epithelial cells, T/NK cells, endothelial cells, monocytes, and fibroblasts. These findings highlight that the lung parenchyma not only supports gas exchange but also maintains a highly organized immune surveillance and tissue-supporting network (**Figure 1D**). Consistent with these compositional differences, visualization of cell distribution patterns in UMAP space revealed clear anatomical preferences among distinct cellular populations (**Figure 1E**). Epithelial cells preferentially localized to the nasal and airway compartments, whereas macrophages and other immune populations were enriched in the lung parenchyma. These results demonstrate that the healthy respiratory system is not composed of a uniform cellular population but instead establishes specialized regional cellular niches adapted to the unique functional requirements of each anatomical compartment.

Given that chronic inflammation represents a central pathological feature of multiple respiratory diseases (Hu et al., 2023; Sin, 2024), we further assessed inflammatory transcriptional characteristics across different cell populations. Analysis of canonical inflammatory genes revealed substantial heterogeneity among cell types, with monocytes exhibiting the strongest expression of inflammation-associated genes (**Figure 1F**). Consistently, calculation of an inflammation score based on inflammatory gene signatures demonstrated that monocytes displayed significantly elevated inflammatory activity compared with other cellular populations (**Figure 1G**, **Table 3**). These findings suggest that circulating and tissue-associated monocytes maintain a distinct inflammatory transcriptional preparedness state even under physiological conditions, potentially enabling rapid responses to environmental challenges or tissue perturbations.

**Table 3:** The genes used in each module score, related to Figure and Figure legends.

Figure 1. Inflammation score	Figure 2.	Figures 6 and 8. Mono_4 subset score
CXCL8	Classical(“CD14”, “S100A8”,”S100A9”, “LYZ”,”FCN1”, “VCAN”,”CTSS”, “CTSD”,”LGALS3”,”CTSL”)	HSPA1B
CCL20	Inflammatory(“IL1B”, “TNF”, “CXCL8”, “CCL3”,”CCL4”, “CXCL2”, “CXCL3”)	CCL20
IL12B	IFN response(“IFIT1”, “IFIT2”,”IFIT3”,”ISG15”, “MX1”,”OAS1”,”OASL”, “IFI6”, “BST2”)	CCL4
CCL3	Non classical(“FCGR3A”,”MS4A7”, “LST1”,”IFITM3”,”IFITM1”,”LILRB1”,”SERPINA1”,”IFITM2”,”SAT1”,”IFITM10”)	CCL3
CCL4	Antigen presentation(“HLA-DRA”, “HLA-DRB1”,”HLA-DPA1”, “HLA-DPB1”,”CD74”, “CIITA”)	CXCL8
CCL5		G0S2
TNF		
IL6		

Collectively, our integrated single-cell atlas reveals pronounced regional cellular specialization and immune microenvironmental heterogeneity across the healthy human respiratory system. Monocytes exhibited prominent inflammatory transcriptional characteristics, providing a reference for subsequent analysis of inflammatory changes in respiratory diseases.

### Identification and functional characterization of HSPA1B^+^ inflammatory monocytes in the healthy human respiratory system.

Monocytes represent a major component of the myeloid immune compartment and play essential roles in tissue surveillance, inflammatory responses, and immune microenvironment remodeling (Hu et al., 2023; Huang et al., 2022). Based on the integrated healthy human respiratory system single-cell transcriptomic atlas described above, we performed high-resolution reclustering analysis of monocyte populations to investigate their transcriptional heterogeneity and functional diversity across different anatomical compartments. Unsupervised clustering identified five transcriptionally distinct monocyte subpopulations (Mono_1–Mono_5 cluster), each exhibiting unique molecular signatures (**Figure 2A**). Differentially expressed gene analysis further demonstrated substantial transcriptional divergence among these subpopulations, with each cluster displaying distinct representative gene expression patterns (**Figure 2B**). These findings indicate that monocytes in the healthy respiratory system comprise heterogeneous functional states rather than a uniform cellular population.

**Figure 2:**
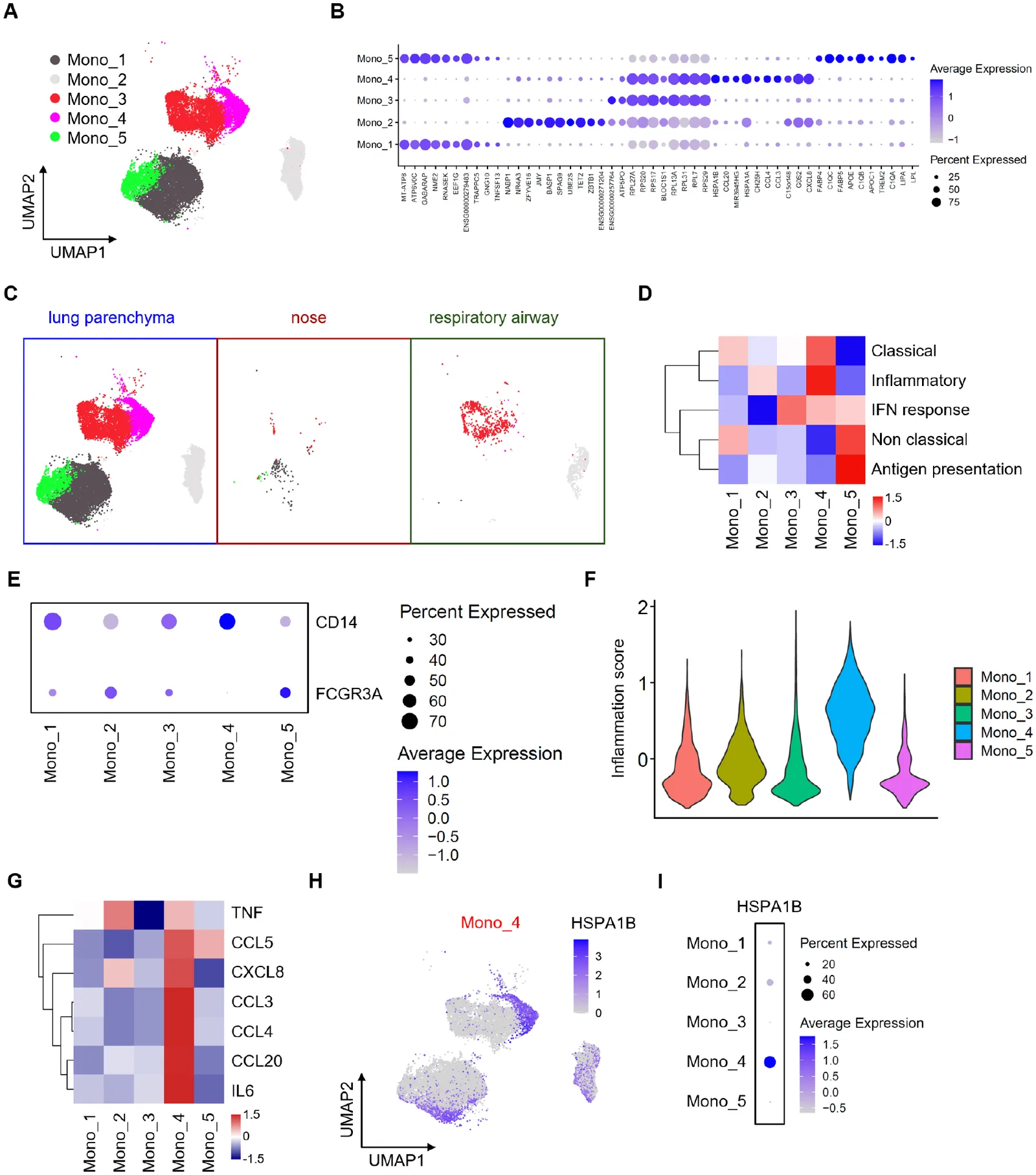
A continuous monocyte state landscape identifies HSPA1B^+^ inflammatory monocytes as a poised inflammatory population in healthy human lungs. **(A)** UMAP visualization of high-resolution monocyte clustering identifies five transcriptionally distinct monocyte states (Mono_1–Mono_5 cluster) in the healthy respiratory system. **(B)** Dot plot displaying representative marker genes defining each monocyte population, highlighting subset-specific transcriptional signatures. **(C)** Tissue distribution of individual monocyte states visualized on UMAP, demonstrating their localization patterns across respiratory compartments. **(D)** Heatmap showing pathway activity scores associated with classical monocyte identity, inflammatory activation, interferon response, non-classical features, and antigen presentation programs across monocyte states. **(E)** Dot plot depicting the expression gradients of CD14 and FCGR3A, defining classical and non-classical monocyte characteristics among identified populations. **(F)** Violin plot showing inflammatory gene signature scores across monocyte subsets. **(G)** Heatmap demonstrating differential expression of inflammatory-associated genes across monocyte subsets. **(H)** Feature plot showing HSPA1B expression across monocyte subsets. **(I)** Dot plot displaying HSPA1B expression across monocyte subsets.

We next examined the anatomical distribution of different monocyte subpopulations across respiratory compartments. Monocytes were predominantly detected in the lung parenchyma, whereas only limited numbers of Mono_2 and Mono_3 clusters were observed in the respiratory airway (**Figure 2C**). This distribution pattern suggests that the lung parenchyma represents a major immune niche supporting monocyte localization and functional regulation under physiological conditions. To further characterize the functional properties of each monocyte subset, we systematically evaluated gene signatures associated with classical monocytes, inflammatory responses, interferon responses, non-classical monocytes, and antigen presentation programs. Mono_4 cluster exhibited the strongest classical monocyte signature accompanied by prominent enrichment of inflammatory gene programs, indicating a highly pro-inflammatory functional state. In contrast, Mono_5 displayed enhanced non-classical monocyte characteristics together with elevated antigen presentation-related signatures, suggesting a potential role in immune surveillance and antigen processing. Mono_1–Mono_3 clusters occupied intermediate positions along the classical-to-non-classical monocyte continuum, exhibiting transitional transcriptional states. Among them, Mono_3 cluster showed increased interferon response signatures, suggesting an interferon-responsive intermediate state (**Figure 2D**, **Table 3**).

Consistent with these functional signatures, analysis of canonical monocyte markers further supported the classification of monocyte subpopulations. Mono_4 cluster selectively expressed high levels of CD14, a hallmark marker of classical monocytes, whereas Mono_5 cluster showed elevated FCGR3A expression, characteristic of non-classical monocytes. Mono_1–Mono_3 cluster exhibited varying degrees of CD14 and FCGR3A expression, further supporting their intermediate phenotypic characteristics along the classical-to-non-classical monocyte spectrum (**Figure 2E**). Given the critical contribution of inflammatory monocytes to respiratory immune responses and chronic inflammatory diseases, we further evaluated inflammatory activity among monocyte subsets. Mono_4 cluster exhibited the highest inflammation score among all monocyte populations, indicating that this subset represents the most prominent inflammatory state within the monocyte compartment (**Figure 2F**). Furthermore, multiple inflammatory mediators, including *TNF*, *CCL5*, *CXCL8*, *CCL3*, *CCL4*, *CCL20*, and *IL6*, were preferentially expressed in Mono_4 cluster compared with other monocyte subsets (**Figure 2G**), further confirming its distinct inflammatory transcriptional profile.

To identify molecular markers associated with this inflammatory monocyte state, we screened genes selectively enriched in Mono_4 cluster and identified HSPA1B as a prominent marker associated with this state. FeaturePlot and DotPlot analyses demonstrated that HSPA1B expression was predominantly restricted to the Mono_4 cluster, supporting its utility as a representative feature of this subset (**Figures 2H, I**). Considering the combined features of high CD14 expression, enrichment of classical monocyte programs, and activation of inflammatory transcriptional signatures, we characterized the Mono_4 cluster as an HSPA1B^+^ inflammatory monocyte state based on its coordinated transcriptional features.

Together, these findings reveal substantial transcriptional heterogeneity among healthy human respiratory monocytes. HSPA1B^+^ inflammatory monocytes formed a distinct subset characterized by classical monocyte features and enhanced expression of inflammatory programs.

### Functional characterization of HSPA1B^+^ inflammatory monocytes and reconstruction of monocyte state transition trajectories

To further define the functional characteristics of HSPA1B^+^ inflammatory monocytes and determine their position within the broader monocyte functional landscape, we performed Gene Ontology (GO) enrichment analysis based on genes specifically upregulated in Mono_4 cluster. The enriched biological processes revealed that HSPA1B^+^ inflammatory monocytes exhibited coordinated activation of inflammatory programs, protein biosynthesis, and cytokine-associated functions (**Figure 3A**). At the Biological Process (BP) level, Mono_4 cluster-specific genes were enriched not only in fundamental cellular processes, including cytoplasmic translation, protein biosynthesis, and translation, but also in inflammatory response pathways, suggesting enhanced biosynthetic activity accompanied by prominent inflammatory transcriptional features. Consistent with these observations, Cellular Component (CC) analysis demonstrated enrichment of Mono_4 cluster-associated genes in ribosome-related structures, including cytosolic large and small ribosomal subunits and ribosomal complexes. In addition, enrichment of extracellular vesicle and secretory granule-associated components suggested involvement in protein production and secretion-related processes. Molecular Function (MF) analysis further revealed significant enrichment of chemokine receptor binding, chemokine activity, cytokine activity, and CCR/CCR5-related ligand–receptor interaction functions (**Figure 3A**). These findings indicate that HSPA1B^+^ inflammatory monocytes possess active chemokine and cytokine signaling programs, consistent with their elevated expression of inflammatory mediators, including TNF, CCL, and CXCL family genes.

**Figure 3:**
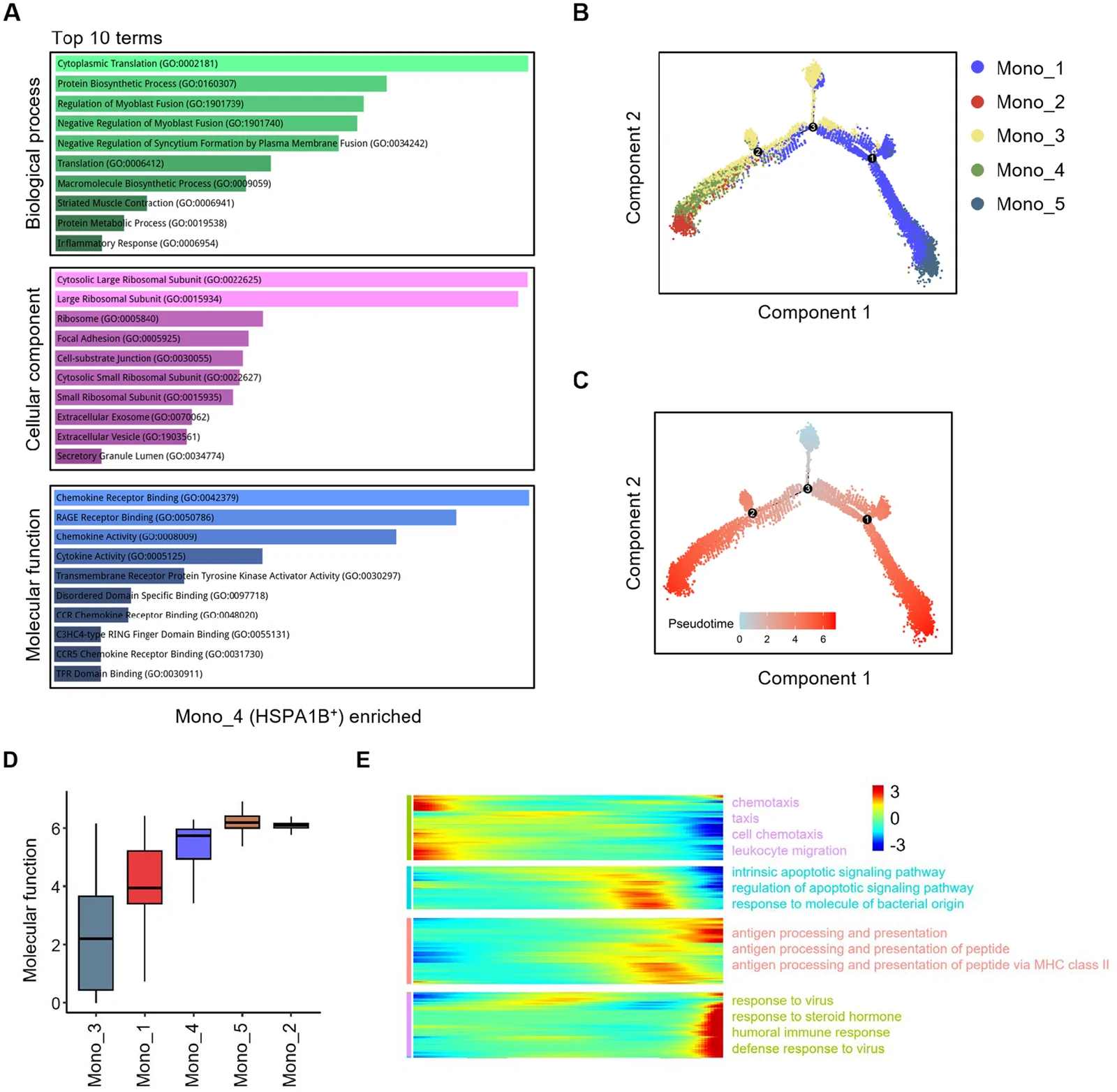
Transcriptional characterization and pseudotime analysis define HSPA1B^+^ inflammatory monocytes within the continuous monocyte landscape. **(A)** Gene Ontology (GO) enrichment analysis of Mono_4 cluster (HSPA1B^+^ inflammatory monocytes)-specific differentially expressed genes, showing significantly enriched biological processes (BP), cellular components (CC), and molecular functions (MF). **(B)** Monocle-based pseudotime analysis illustrating the inferred transcriptional ordering and branch structure among monocyte subsets. **(C)** Pseudotime ordering of monocyte populations showing the distribution of transcriptional states along the inferred trajectory. **(D)** Distribution of monocyte subsets along pseudotime trajectory. **(E)** Gene Ontology enrichment analysis of pseudotime-dependent differentially expressed genes.

To investigate the transcriptional relationships and potential ordering of monocyte subpopulations, we constructed pseudotime trajectories based on single-cell transcriptomic profiles (**Figures 3B–D**). Monocyte subsets were continuously distributed along the pseudotime trajectory rather than forming isolated clusters, suggesting the existence of a dynamic transcriptional continuum representing different functional states. Trajectory analysis revealed that Mono_3 cluster occupied a position with relatively low pseudotime values, indicating that this population was positioned earlier in the inferred transcriptional ordering (**Figures 3C, D**). The trajectory structure further separated monocyte states into two branches, with one branch associated with Mono_4 and Mono_2 and the other extending through Mono_1 toward Mono_5. The branch associated with Mono_4 and Mono_2 showed inflammatory and classical monocyte features, whereas the Mono_1–Mono_5 branch was characterized by non-classical monocyte and antigen presentation-related features (**Figures 3B, C**). These findings suggest that healthy respiratory monocytes display continuous transcriptional variation across distinct cellular states rather than forming strictly discrete classical and non-classical populations.

To further characterize functional changes associated with the inferred pseudotime ordering, we analyzed differentially expressed genes across pseudotime stages and performed functional enrichment analysis (**Figure 3E**). Genes associated with lower pseudotime values were primarily associated with chemotaxis, leukocyte migration, and cell trafficking-related programs, indicating enrichment of tissue trafficking-related transcriptional programs at this position in the inferred ordering. Across the inferred trajectory, immune activation-related pathways, including responses to bacterial-derived molecules and regulation of intrinsic apoptotic signaling, became increasingly enriched, indicating changes in immune-related transcriptional programs across the inferred ordering. Distinct pseudotime regions showed enrichment of distinct immune-related programs. One trajectory branch showed enrichment of antigen processing and presentation pathways, including MHC class II-mediated peptide presentation, consistent with an antigen presentation-associated transcriptional state. Other regions of the trajectory displayed enhanced antiviral responses, responses to external stimuli, and immune regulatory programs, indicating transcriptional specialization among distinct monocyte states.

Taken together, these analyses reveal continuous transcriptional variation among monocyte states in the healthy human respiratory system. HSPA1B^+^ inflammatory monocytes were characterized by enhanced inflammatory and biosynthetic programs, whereas other monocyte states showed distinct immune-related transcriptional features.

### Cell–cell communication networks in healthy lung tissue reveal monocyte-mediated immune regulation.

To systematically characterize the intercellular communication landscape within the healthy human lung microenvironment, we constructed a ligand–receptor interaction-based cell–cell communication network incorporating major lung cell populations, including endothelial cells, fibroblasts, dendritic cells, T/NK cells, other immune cells, epithelial cells, B/plasma cells, macrophages, and five monocyte subpopulations (Mono_1–Mono_5 cluster) (**Figures 4A, B**). Global network analysis revealed that healthy lung tissue exhibited an interconnected pattern of predicted cell–cell signaling interactions. Among all cellular populations, fibroblasts, macrophages, and monocytes exhibited prominent predicted communication connectivity and signaling activity, suggesting that structural stromal cells and myeloid immune populations represent major nodes of predicted signaling interactions within the lung microenvironment. Fibroblasts primarily contributed to extracellular matrix (ECM)-associated signaling involved in tissue structural maintenance, whereas myeloid populations participated in immune regulatory networks associated with immune-related signaling programs. Among immune populations, monocytes displayed extensive bidirectional communication patterns, forming multiple interaction networks with fibroblasts, epithelial cells, endothelial cells, and dendritic cells. These findings suggest that monocytes are not merely circulating immune effector cells but are associated with diverse signaling interactions across tissue compartments in the healthy lung microenvironment.

**Figure 4:**
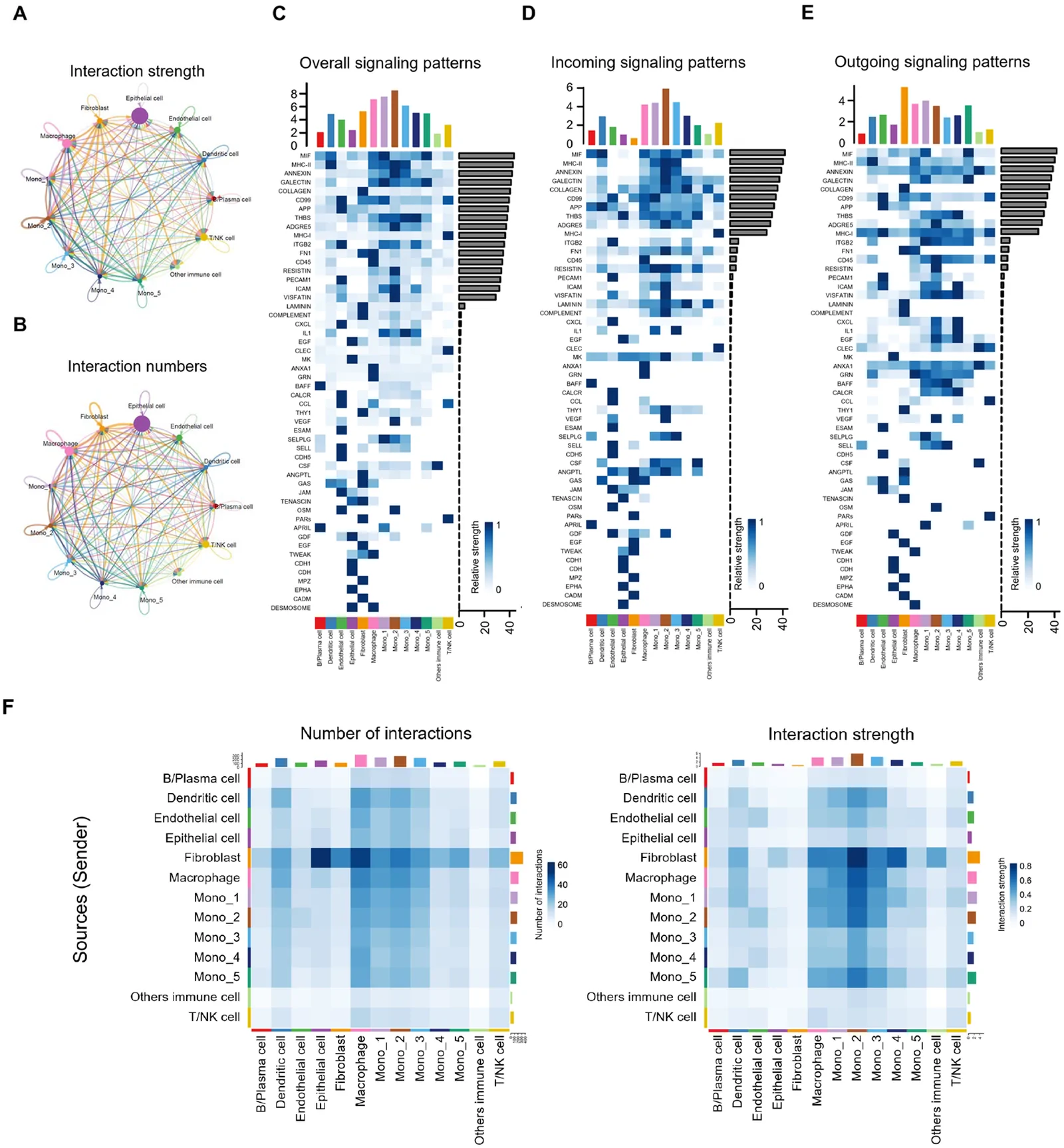
Cell-cell communication profiling reveals a coordinated stromal-immune signaling patterns in the healthy respiratory system. **(A, B)** Global cell–cell communication networks within the healthy human respiratory system were inferred using CellChat. Interaction strength **(A)** and interaction number **(B)** were calculated to characterize communication connectivity among major annotated cell populations, including epithelial cells, endothelial cells, fibroblasts, macrophages, dendritic cells, B/plasma cells, T/NK cells, other immune cells, and distinct monocyte subsets (Mono_1–Mono_5 cluster). **(C)** Heatmap depicting the overall landscape of signaling pathway activities across different respiratory cell populations. **(D)** Heatmap illustrating incoming signaling patterns, highlighting the major receiver populations and their regulatory inputs within the intercellular communication network. **(E)** Heatmap showing outgoing signaling profiles, representing dominant signal-sending populations and their contribution to respiratory tissue communication. **(F)** Quantitative comparison of intercellular communication patterns based on the number and strength of inferred ligand–receptor interactions across cell populations.

We further examined communication patterns among distinct monocyte subpopulations. All Mono_1–Mono_5 clusters participated in interactions with multiple cellular compartments, indicating that monocyte heterogeneity represents different functional states embedded within a shared communication ecosystem rather than isolated cellular populations. Distinct monocyte subsets exhibited variable signal receiving and transmitting capacities, suggesting specialized roles in their respective signaling profiles within healthy lung tissue. To identify dominant signaling programs shaping the healthy lung communication network, we performed global signaling pattern analysis (**Figure 4C**). Three major functional communication modules were identified, including extracellular matrix maintenance, immune surveillance, and barrier-associated regulation. ECM-related signaling pathways, including COLLAGEN, LAMININ, and FN1, were primarily mediated by fibroblasts and other structural cells, forming a fundamental communication framework supporting lung tissue architecture and immune cell niches. In parallel, immune regulatory pathways, including MIF, ANNEXIN, and GALECTIN, showed substantial information flow contributions, indicating that healthy lung tissue maintains active immune-related signaling programs rather than a completely quiescent immune state.

Furthermore, immune-related communication programs involving IL1 signaling, MHC-I/II antigen presentation, ITGB2-mediated immune adhesion, and complement pathways were predominantly associated with macrophage and monocyte populations, establishing a myeloid-centered immune regulatory network (**Figure 4C**). Although epithelial and endothelial cells are not classical immune populations, they also contributed to CXCL-, MHC-, and adhesion molecule-associated signaling, highlighting extensive signaling interactions among immune, barrier, and vascular compartments within the healthy respiratory microenvironment. Analysis of incoming signaling activity demonstrated that myeloid populations represented major signal receivers in healthy lung tissue. In particular, Mono_2 cluster and macrophages exhibited strong incoming signaling activity and received diverse signaling inputs involving MIF, ANNEXIN, GALECTIN, COLLAGEN, and MHC-related pathways (**Figure 4D**). These results suggest that tissue-adapted myeloid populations continuously integrate immune, stromal, and epithelial-derived cues to maintain functional adaptation and immune surveillance. Conversely, outgoing signaling analysis identified fibroblasts as dominant signal sources, mainly through ECM-associated pathways including COLLAGEN, LAMININ, and FN1 signaling. Meanwhile, macrophages and monocyte subsets demonstrated strong immune-related outgoing activity mediated by MIF, CXCL, ANNEXIN, and GALECTIN signaling, forming a coordinated myeloid-centered communication module (**Figure 4E**). Together, these findings reveal a hierarchical stromal–immune communication framework in which fibroblasts provide structural niche-associated signals, whereas monocytes and macrophages regulate immune information exchange within the lung microenvironment.

Although interaction numbers and signaling strength showed partial consistency, different cell populations exhibited distinct functional contributions within the communication network. Fibroblasts and macrophages displayed both high interaction numbers and strong signal intensity, consistent with their roles as major structural and immune regulatory signaling populations (**Figure 4F**). Interestingly, certain monocyte subsets, particularly Mono_1 and Mono_2 cluster, exhibited relatively fewer interaction partners but maintained strong signaling intensity, suggesting that these populations may exert distinct signaling activity through a relatively limited set of ligand–receptor interactions rather than extensive communication networks (**Figure 4F**).

Taken together, the CellChat analysis identified extensive predicted signaling interactions among stromal and immune populations in healthy lung tissue. Fibroblasts showed prominent ECM-associated signaling, while macrophages and monocyte subsets were involved in MIF-, CXCL-, adhesion-, and other immune-related signaling pathways.

### HSPA1B^+^ inflammatory monocytes represent key communication nodes within the healthy respiratory immune network

To further delineate the regulatory architecture underlying intercellular communication within the healthy respiratory microenvironment, we performed an information flow analysis of major signaling pathways inferred by CellChat (**Figure 5A**). Among all evaluated signaling programs, MIF signaling exhibited the highest overall information flow, indicating its predominant contribution to maintaining communication networks across respiratory cell populations. In addition, several other signaling pathways, including ANNEXIN, GALECTIN, COLLAGEN, MHC-I, ITGB2, FN1, and THBS, displayed substantial communication activity (**Figure 5A**). These highly active signaling programs collectively represent diverse biological processes, including immune regulation, extracellular matrix (ECM) sensing, cell adhesion, and tissue structural maintenance, suggesting that the healthy respiratory system establishes a coordinated communication landscape through the integration of immune surveillance and tissue-supportive signaling programs.

**Figure 5:**
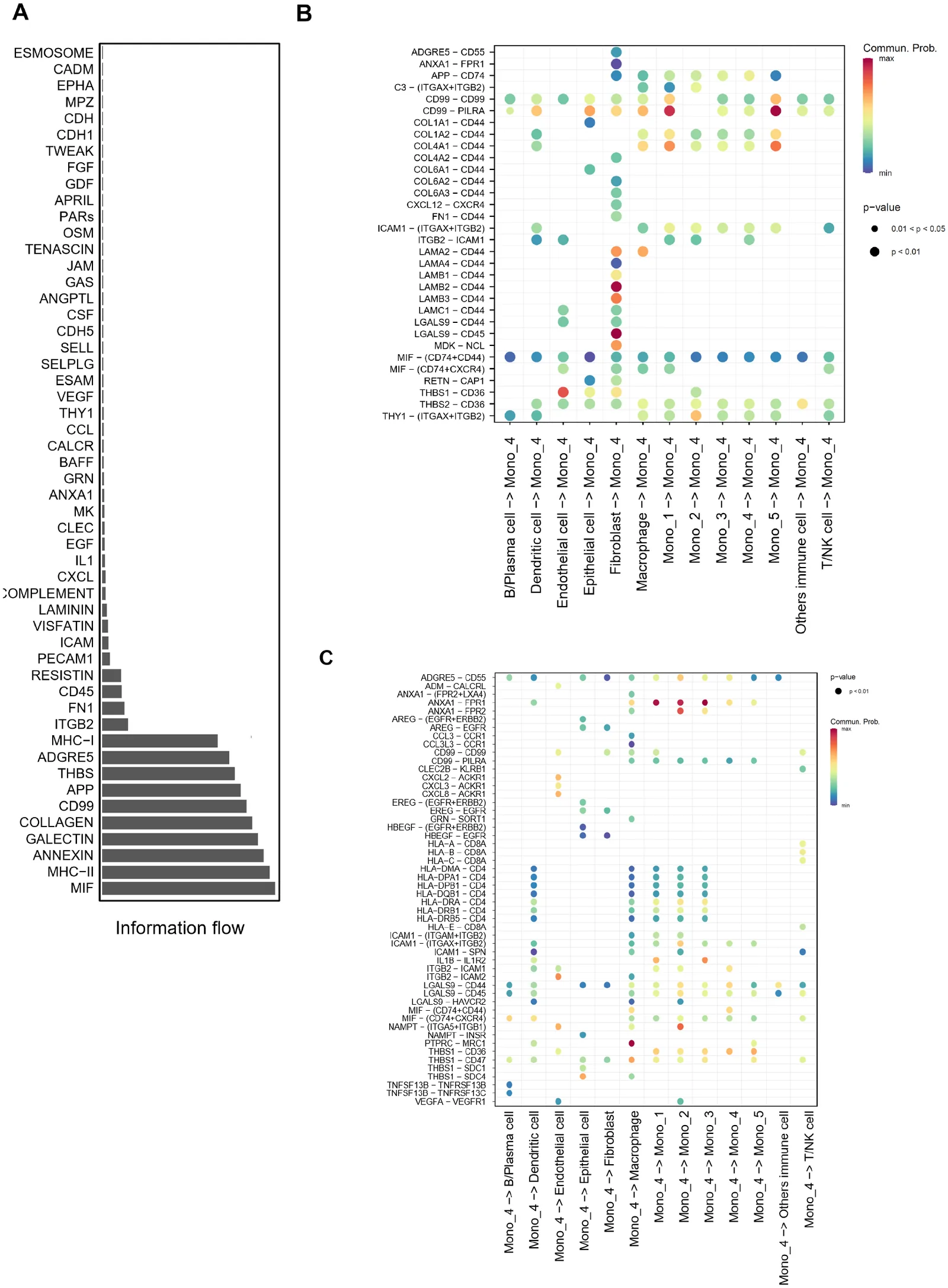
Dominant signaling programs and ligand–receptor interactions associated with HSPA1B^+^ inflammatory monocytes in the healthy respiratory system. **(A)** Information flow analysis of major signaling pathways across the respiratory system communication network. **(B)** Dot plot of ligand-receptor interactions between Mono_4 cluster (HSPA1B^+^ inflammatory monocytes) and other cell types. Mono_4 cluster is shown as the signal receiver. **(C)** Dot plot of ligand-receptor interactions between Mono_4 cluster (HSPA1B^+^ inflammatory monocytes) and other cell types. Mono_4 cluster functions as an active signaling source.

Given the prominent inflammatory signature of the Mono_4 cluster (HSPA1B^+^ inflammatory monocytes), we next investigated its communication characteristics within the respiratory cellular ecosystem. Ligand–receptor interaction analysis revealed that Mono_4 exhibited active bidirectional communication properties, characterized by extensive signal reception from surrounding cell populations and concurrent signal transmission to neighboring cells (**Figures 5B, C**). These findings suggest that Mono_4 cluster may function as an interactive immune population capable of integrating environmental cues and participating in local immune regulation. As a signal-receiving population, Mono_4 cluster was influenced by diverse signals originating from both immune and structural cell populations. Among these interactions, MIF-associated signaling represented one of the most prominent incoming communication programs, primarily mediated through MIF-(CD74+CD44) and MIF-(CD74+CXCR4) ligand–receptor pairs. The convergence of MIF signals derived from multiple cellular sources onto shared receptor systems suggests a potential mechanism through which tissue-derived cues may regulate Mono_4 functional states. In addition, Mono_4 received multiple ECM- and adhesion-related signals, including COL1A1/COL1A2/COL4A1-CD44, LAMA-CD44, LGALS9-CD45, THBS1-CD36, and ITGB2-ICAM1 interactions, indicating that structural components and immune-associated adhesion pathways may contribute to shaping the functional characteristics of inflammatory monocytes within healthy lung tissue (**Figure 5B**).

Conversely, Mono_4 cluster also acted as a signal-sending population capable of communicating with surrounding cells through multiple immune regulatory pathways. Mono_4-derived MIF-(CD74+CD44) and MIF-(CD74+CXCR4) interactions suggested potential involvement in myeloid cell communication networks. Furthermore, Mono_4 cluster displayed communication with vascular-associated cells through chemokine-related interactions, including CXCL8-ACKR1, CXCL3-ACKR1, and CXCL2-ACKR1, suggesting a potential role in modulating endothelial-associated immune trafficking programs. Additional signaling axes, including ANXA1-FPR1/FPR2, THBS1-CD36/CD47, and ICAM1-ITGB2, further highlighted the capacity of Mono_4 cluster to participate in inflammatory regulation, cellular adhesion, and tissue–immune interactions (**Figure 5C**).

These analyses showed that HSPA1B^+^ inflammatory monocytes exhibited relatively strong predicted incoming and outgoing signaling activity and were involved in multiple MIF, CXCL, and adhesion-related ligand–receptor interactions. These findings characterize the inferred signaling profile of HSPA1B^+^ inflammatory monocytes within the healthy respiratory microenvironment.

### Increased representation and disease-associated features of HSPA1B^+^ inflammatory monocytes in COPD lungs

To determine whether the HSPA1B^+^ inflammatory monocytes state identified in healthy respiratory tissues is associated with chronic inflammatory lung disease, we integrated single-cell RNA sequencing datasets from healthy individuals and patients with chronic obstructive pulmonary disease (COPD) to characterize disease-associated alterations in cellular composition and monocyte functional states. Unsupervised clustering combined with canonical marker gene expression identified 11 major cell populations across healthy and COPD lung tissues, including B/plasma cells, dendritic cells, endothelial cells, epithelial cells, fibroblasts, macrophages, mast cells, monocytes, neutrophils, other immune cells, and T/NK cells (**Figure 6A**, **Table 2**). Marker gene expression analysis further confirmed the accuracy of cell annotation, with each cellular population exhibiting distinct molecular signatures (**Figure 6B**). Dimensionality reduction analysis based on individual samples demonstrated substantial mixing of cells from different donors without obvious sample-specific clustering patterns, indicating robust data integration and suitability for comparative analysis of disease-associated cellular remodeling (**Figure 6C**).

**Figure 6:**
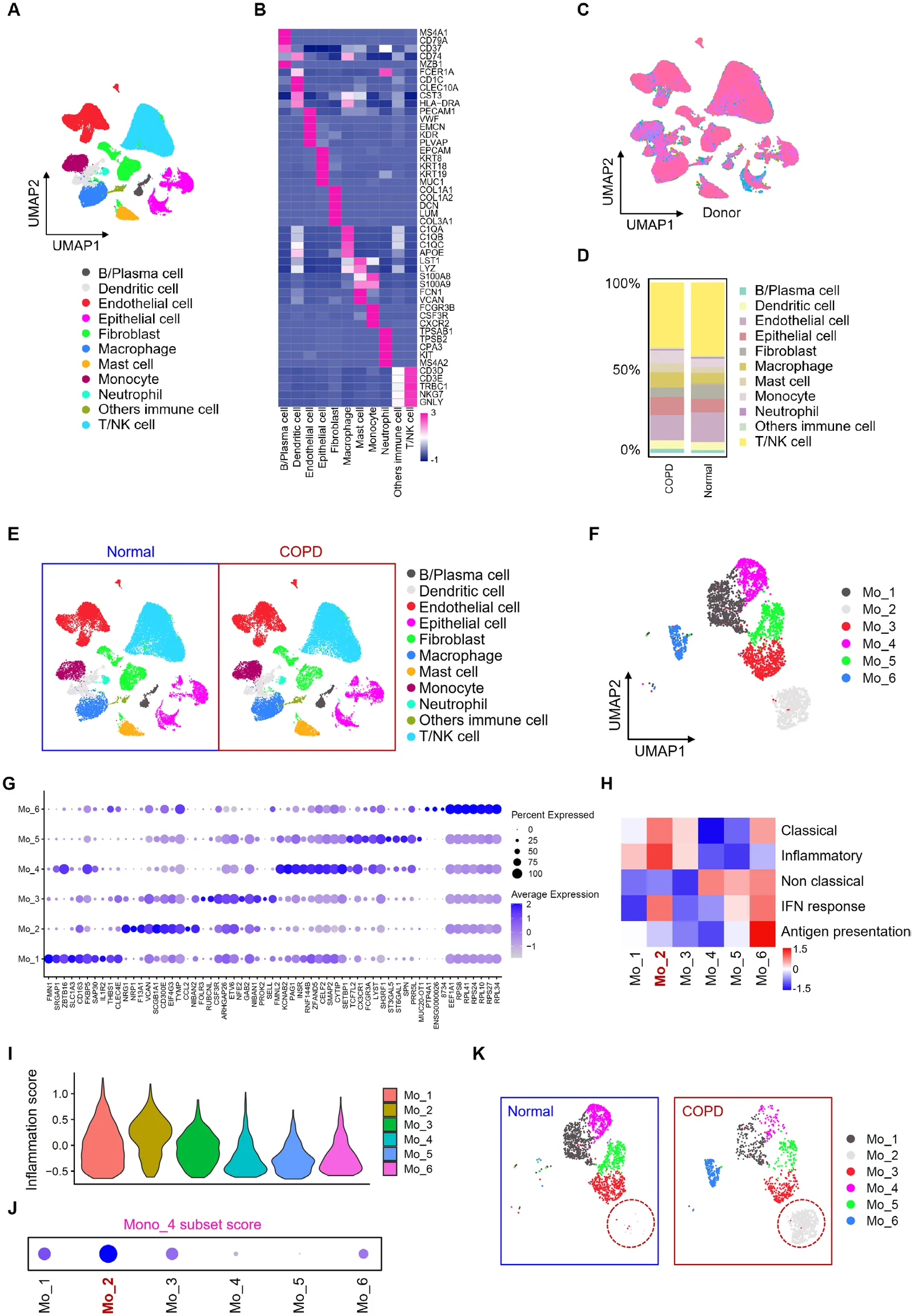
Increased representation of an HSPA1B^+^ inflammatory monocytes state in COPD lungs. **(A)** UMAP visualization of the integrated single-cell transcriptomic landscape of human lung tissues from healthy donors (n = 7) and COPD (n = 9) patients. Major cellular populations were identified based on canonical marker expression, including B/plasma cells, dendritic cells, endothelial cells, epithelial cells, fibroblasts, macrophages, mast cells, monocytes, neutrophils, other immune cells, and T/NK cells. **(B)** Heatmap depicting the expression profiles of canonical marker genes supporting the annotation of major cell populations. **(C)** UMAP embedding illustrating the contribution of individual samples from healthy donors and COPD patients, demonstrating effective integration of datasets with minimal sample-associated segregation. **(D)** Comparison of cellular composition across lung tissues from healthy donors and COPD patients, showing the relative proportion of major cell populations. **(E)** UMAP visualization displaying the distribution patterns of annotated cell populations in healthy and COPD lung tissues. **(F)** Re-clustering of monocyte populations into six subsets (Mo_1-Mo_6 clusters). **(G)** Bubble plot showing expression of marker genes across different monocyte subsets. **(H)** Heatmap showing pathway activity scores associated with classical monocyte identity, inflammatory activation, interferon response, non-classical features, and antigen presentation programs across different monocyte subsets. **(I)** Violin plot showing inflammatory gene signature scores across different monocyte subsets. **(J)** Bubble plot showing the gene signature scores of Mono_4 cluster (HSPA1B^+^) across different monocyte subsets. **(K)** UMAP visualization displaying the distribution patterns of monocyte subsets in healthy (n = 7) and COPD (n = 9) lung tissues.

We next compared the cellular composition between healthy and COPD lung tissues. COPD lungs exhibited marked alterations in the immune microenvironment, characterized by increased proportions of monocytes, macrophages, mast cells, and epithelial cells, accompanied by reduced proportions of T/NK cells and fibroblasts compared with healthy lungs (**Figure 6D**). These findings indicate that COPD progression is associated with profound remodeling of the pulmonary cellular ecosystem, particularly involving expansion and functional alteration of myeloid immune populations. Consistent with these compositional changes, visualization of cellular distribution patterns in UMAP space revealed disease-associated redistribution of multiple immune populations. Notably, monocyte- and macrophage-enriched regions were expanded in COPD lungs, suggesting substantial myeloid compartment remodeling during chronic inflammatory disease progression (**Figure 6E**).

Given the prominent inflammatory heterogeneity of monocytes observed in healthy lung tissue, we performed high-resolution reclustering of COPD-derived monocytes and identified six transcriptionally distinct monocyte subsets (Mo_1–Mo_6 clusters) (**Figure 6F**). Differential gene expression analysis demonstrated distinct molecular programs among these subsets, confirming functional heterogeneity within COPD-associated monocyte populations (**Figure 6G**). To define the functional characteristics of COPD monocyte states, we evaluated classical monocyte, inflammatory response, non-classical monocyte, interferon response, and antigen presentation-related gene signatures. Mo_1–Mo_3 clusters predominantly exhibited classical monocyte characteristics, whereas Mo_4–Mo_6 clusters displayed stronger non-classical monocyte features (**Figure 6H**). Among these populations, Mo_2 cluster demonstrated the strongest inflammatory signature and showed enhanced interferon response activity under COPD conditions, suggesting acquisition of a disease-associated activated inflammatory state. Consistently, inflammation score analysis revealed that Mo_2 cluster exhibited the highest inflammatory activity among all COPD monocyte subsets (**Figure 6I**), further supporting its identity as a dominant inflammatory monocyte population within the COPD lung microenvironment.

To determine whether COPD-associated Mo_2 cluster represented a disease-associated counterpart of the healthy HSPA1B^+^ inflammatory monocytes state, we performed cross-condition transcriptional state comparison using the characteristic gene signature derived from healthy lung Mono_4 cluster (HSPA1B^+^ inflammatory monocytes). Strikingly, the HSPA1B^+^ inflammatory monocytes signature was most strongly enriched in Mo_2 cluster, demonstrating substantial transcriptional similarity between COPD Mo_2 cluster and healthy HSPA1B^+^ inflammatory monocytes (**Figure 6J**, **Table 3**). These results suggest that Mo_2 cluster represents a distinct HSPA1B^+^ inflammatory monocyte state observed in the analyzed respiratory samples rather than an entirely novel inflammatory population emerging during COPD progression. Further comparison of this inflammatory monocyte state between healthy and COPD lungs revealed a trend toward greater representation of Mo_2-like HSPA1B^+^ inflammatory monocytes in COPD tissues, accompanied by differences in their representation within UMAP space (**Figure 6K**). These findings indicate that COPD-associated inflammation is accompanied by altered representation and functional features of an HSPA1B^+^ inflammatory monocyte state, with a trend toward greater representation in COPD lungs.

Taken together, these findings reveal substantial immune and transcriptional remodeling in COPD lungs, including increased representation of an HSPA1B^+^ inflammatory monocyte-like state. This state showed prominent inflammatory and interferon-associated transcriptional features, linking the HSPA1B^+^ inflammatory monocyte program to the altered immune landscape of COPD lungs.

### m^6^A regulatory profiling reveals WTAP-associated inflammatory programs in monocyte functional states.

Epitranscriptomic regulation, particularly N6-methyladenosine (m^6^A) modification, represents an important regulatory layer controlling immune cell differentiation, functional plasticity, and inflammatory responses (Cai et al., 2025; Du et al., 2026; Yang J. et al., 2026). To investigate whether m^6^A regulatory programs are associated with monocyte functional states, we systematically analyzed the expression patterns of m^6^A-associated regulators across different monocyte subpopulations. Unsupervised clustering based on whole-transcriptome profiles and m6A regulatory gene expression patterns revealed that m^6^A-related regulators alone were sufficient to distinguish distinct monocyte functional states, showing comparable or even enhanced discriminatory capacity compared with global transcriptional profiles (**Figures 7A, B**). These findings suggest that m^6^A regulatory signatures are closely coupled with monocyte functional identities and may represent an epitranscriptional feature associated with immune state specification.

**Figure 7:**
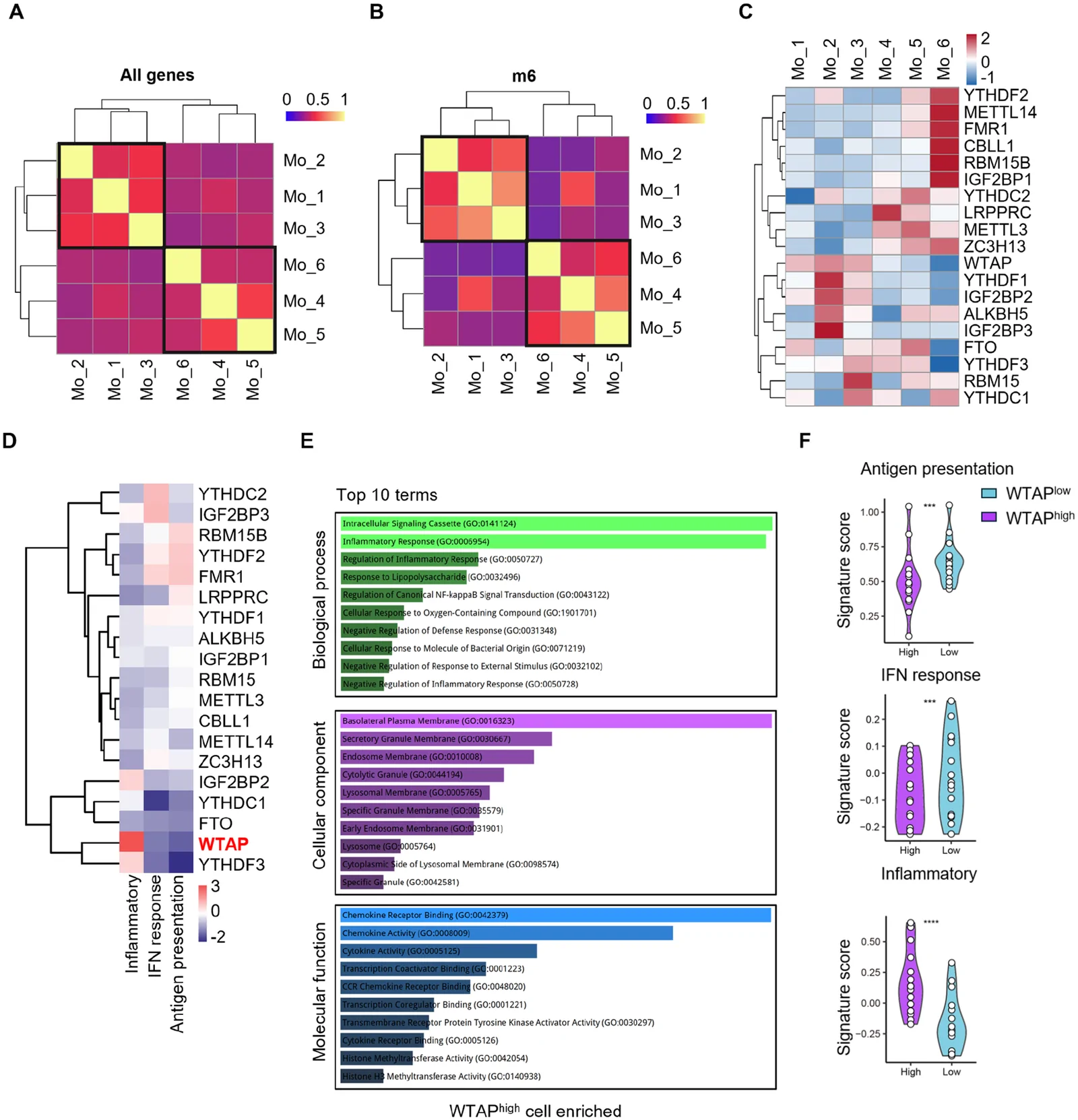
WTAP-associated m^6^A regulatory programs define inflammatory monocyte state in COPD lungs. **(A)** Unsupervised clustering of monocyte subsets based on genome-wide transcriptomic profiles. **(B)** Unsupervised clustering of monocyte subsets using the expression patterns of m^6^A regulatory genes. **(C)** Heatmap illustrating the heterogeneous expression landscape of m^6^A -associated regulators across monocyte subsets. **(D)** Correlation analysis linking m^6^A regulator expression with monocyte functional programs, including inflammatory activation, interferon response, and antigen presentation signatures. **(E)** GO enrichment analysis of genes upregulated in WTAP-high monocytes. **(F)** Comparison of inflammatory activation, interferon response, and antigen presentation gene programs between WTAP-high and WTAP-low monocytes. Each dot represents one donor (n = 16). Statistical significance was assessed using a two-tailed paired Wilcoxon signed-rank test **(F)**. *P < 0.05; ***P < 0.001; ****P < 0.0001.

We next characterized the expression landscape of major m^6^A regulatory components, including methyltransferase “writers” (METTL3, METTL14, WTAP), demethylase “erasers” (ALKBH5 and FTO), and RNA-binding “readers” (YTHDF1–3 and YTHDC1–2). Distinct monocyte populations exhibited differential expression patterns of these regulators, indicating that the m^6^A regulatory machinery undergoes dynamic remodeling across monocyte functional states rather than representing a fixed regulatory module (**Figure 7C**). To determine the relationship between m^6^A regulators and monocyte functional programs, we performed correlation analysis between regulator expression and functional signatures. Among the analyzed regulators, WTAP expression displayed the strongest association with inflammatory characteristics, showing a positive correlation with inflammation scores and an inverse correlation with interferon response and antigen presentation signatures (**Figure 7D**). These results suggest that WTAP expression is closely linked to the functional balance between inflammatory execution programs and immune surveillance-associated states.

Given the strong association between WTAP and inflammatory monocyte characteristics, we further investigated the transcriptional features of WTAP-high monocytes. Gene Ontology enrichment analysis of genes upregulated in WTAP-high monocytes demonstrated significant enrichment of inflammatory and innate immune activation programs, including inflammatory response, regulation of inflammatory response, response to lipopolysaccharide, and regulation of canonical NF-κB signaling pathways (**Figure 7E**). In addition, WTAP-high monocytes exhibited enhanced transcriptional programs associated with cellular responses to bacterial-derived stimuli and intracellular signaling regulation, suggesting a close relationship between WTAP-associated states and inflammatory signal amplification. Cellular Component analysis revealed that WTAP-high monocyte-associated genes were enriched in immune effector structures, including secretory granule membranes, lysosomal membranes, endosomal membranes, and cytotoxic granule-associated compartments. These findings suggest enhanced intracellular trafficking, protein processing, and inflammatory mediator release capacity in WTAP-high monocytes. Furthermore, Molecular Function analysis demonstrated enrichment of chemokine receptor binding, chemokine activity, cytokine activity, cytokine receptor binding, and CCR-associated chemokine functions (**Figure 7E**), indicating increased potential for chemokine production and immune cell communication. Together, these results indicate that WTAP-high monocytes represent a distinct inflammatory functional state rather than simply reflecting generalized inflammatory gene elevation. Instead, this population exhibits coordinated activation of inflammatory signaling, chemokine production, and immune-related transcriptional programs, consistent with a WTAP-associated inflammatory state.

To further validate the association between WTAP expression and monocyte functional states, we compared functional signatures between WTAP-high and WTAP-low monocytes. WTAP-high monocytes displayed significantly increased inflammation scores, whereas antigen presentation and interferon response signatures were markedly reduced (**Figure 7F**). These findings suggest that WTAP-associated monocytes exhibit distinct inflammatory and immune surveillance-related transcriptional programs. Collectively, these analyses identify WTAP expression as a feature associated with inflammatory monocyte states. WTAP-high monocytes showed enhanced inflammatory transcriptional programs, including NF-κB-associated and chemokine/cytokine-related signatures, providing a basis for further investigation of WTAP-associated regulation in inflammatory monocytes.

### WTAP is associated with enhanced inflammatory and reduced interferon-responsive and antigen-presentation programs in monocytes.

To investigate whether WTAP is functionally associated with the inflammatory transcriptional state, we performed loss-of-function experiments using a human monocyte-derived inflammatory activation model. THP-1 monocytes were first transfected with WTAP-specific siRNA, followed by stimulation with lipopolysaccharide (LPS) and interferon-γ (IFN-γ) to induce an inflammatory monocyte-like state, as illustrated in **Figure 8A**. The flow cytometric analysis confirmed a decrease in WTAP^+^ cells following WTAP knockdown, while cell viability remained above 90% (**Figure 8B**). Notably, WTAP knockdown also significantly reduced *HSPA1B* expression following inflammatory stimulation (**Figure 8C**), providing additional evidence linking WTAP-associated transcriptional changes to the HSPA1B^+^ inflammatory monocyte program. Transcriptomic evaluation revealed that WTAP silencing was associated with changes in monocyte functional programs. Specifically, knockdown of WTAP markedly reduced the expression of the WTAP, canonical inflammatory genes, including *IL1B*, *TNF*, *CXCL8*, and *IL6* (**Figure 8C**), indicating attenuation of the inflammatory activation program. In contrast, WTAP depletion significantly enhanced the expression of genes associated with interferon-responsive pathways, including *ISG15*, *IFIT1*, *IFIT3*, *MX1*, *OAS1*, and *RSAD2*, as well as genes involved in antigen processing and presentation, including *HLA-DRA*, *HLA-DRB1*, *CD74*, *CIITA*, *HLA-DPA1*, and *CTSS* (**Figure 8C**).

**Figure 8:**
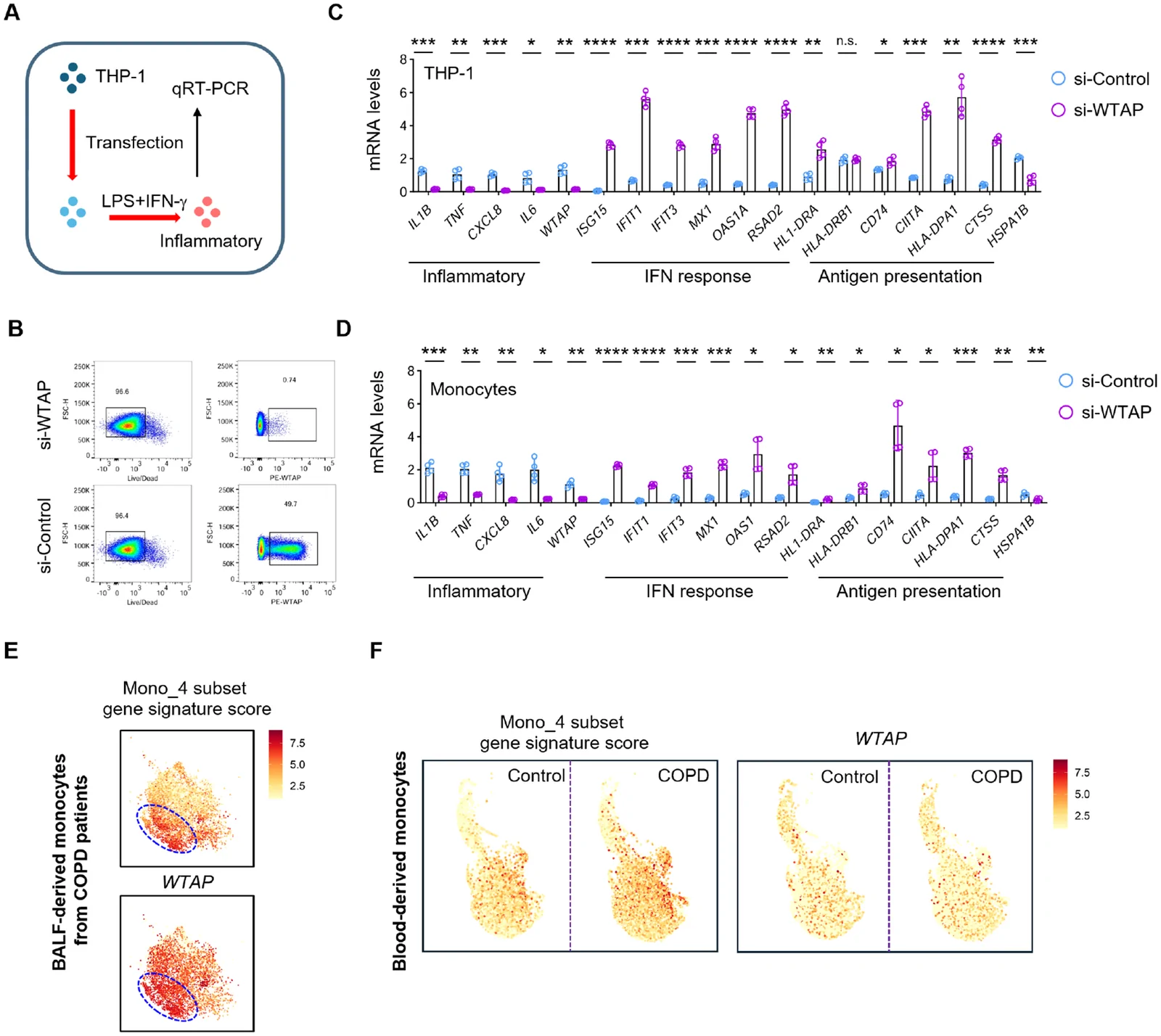
WTAP shapes inflammatory monocyte state by enhancing inflammatory programs and limiting alternative immune programs. **(A)** Schematic overview of the experimental design. Human THP-1 monocytes were transfected with WTAP-specific siRNA or control siRNA, followed by stimulation with lipopolysaccharide (LPS) and interferon-γ (IFN-γ) to induce an inflammatory activation state. **(B)** Flow cytometric analysis of WTAP protein expression and cell viability in control siRNA (si-Control) and WTAP siRNA (si-WTAP) transfected THP-1 cells. **(C)** qRT-PCR analysis evaluating the expression of the WTAP, HSPA1B, inflammatory, interferon-responsive, and antigen-presentation-related gene programs in control siRNA (si-Control, n = 4) and WTAP siRNA (si-WTAP, n = 4) transfected THP-1 cells. **(D)** qRT-PCR analysis evaluating the expression of WTAP, HSPA1B, inflammatory, interferon-responsive, and antigen-presentation-related genes in control siRNA (si-Control, n = 4) and WTAP siRNA (si-WTAP, n = 4) transfected primary human monocytes. **(E)** UMAP visualization of BALF-derived monocyte subsets from COPD patients, showing the expression of HSPA1B^+^ inflammatory monocytes signature (Mono_4 cluster signature) and WTAP. **(F)** UMAP visualization of peripheral blood monocyte subsets from healthy individuals and COPD patients, showing the expression of HSPA1B^+^ inflammatory monocytes signature and WTAP. n.s., no significance; *P < 0.05; **P < 0.01; ***P < 0.001; ****P < 0.0001 (two-tailed Students’ t test in **C, D**).

To further validate whether the WTAP-associated transcriptional changes observed in the THP-1 model were also relevant to primary human monocytes, we performed a complementary experiment using primary human monocytes (**Figure 8D**). Consistent with the findings in THP-1 cells, WTAP knockdown in primary human monocytes significantly reduced HSPA1B expression and the expression of inflammatory genes, including *IL1B*, *TNF*, *CXCL8*, and *IL6*, while altering representative interferon-responsive and antigen-presentation-related genes. These findings provide additional evidence that the WTAP-associated transcriptional features observed in the THP-1 model are also detectable in primary human monocytes and further support the association between WTAP and the HSPA1B^+^ inflammatory monocyte program.

To determine whether the WTAP-associated inflammatory program identified *in vitro* corresponds to disease-associated monocyte states *in vivo*, we further analyzed single-cell transcriptomic datasets from bronchoalveolar lavage fluid (BALF) of COPD patients. Unsupervised clustering of BALF-derived monocytes identified distinct monocyte populations, and projection of the healthy lung-derived HSPA1B^+^ inflammatory monocytes signature (Mono_4 cluster signature) revealed a preferential enrichment within a specific monocyte subset in COPD samples (**Figure 8E**, **Tables 2** and **3**). Notably, this population exhibited significantly elevated WTAP expression, demonstrating a strong concordance between WTAP expression and the disease-associated inflammatory monocyte program. These results suggest that WTAP-associated inflammatory features are preserved in COPD airway inflammatory environments. To further determine whether this inflammatory monocyte state represents a circulating precursor population, we analyzed peripheral blood monocyte scRNA-seq datasets from healthy individuals and COPD patients. In contrast to BALF-derived monocytes, peripheral blood monocytes from both healthy controls and COPD patients showed limited enrichment of the Mono_4 cluster signature (HSPA1B^+^ inflammatory monocytes), accompanied by relatively low WTAP expression levels (**Figure 8F**, **Tables 2** and **3**). This finding suggests that the WTAP-associated inflammatory monocyte state is preferentially established within the lung inflammatory niche rather than broadly expanded in circulating monocytes. Collectively, these results identify WTAP as a factor associated with the COPD-associated inflammatory monocyte program. WTAP perturbation is associated with reduced inflammatory effector gene expression and increased interferon-responsive and antigen-presentation-related transcriptional programs, providing functional evidence for a WTAP-associated inflammatory transcriptional program in monocytes.

In summary, these findings link WTAP expression to the inflammatory transcriptional state of HSPA1B^+^ inflammatory monocytes. WTAP depletion reduced inflammatory gene expression while enhancing interferon-response and antigen-presentation-related programs, indicating that WTAP-associated transcriptional regulation is linked to the inflammatory phenotype of these monocytes. Together with the single-cell and COPD transcriptomic analyses, these results support an association between the WTAP-associated monocyte program and the inflammatory cellular landscape observed in COPD (**Figure 9**).

**Figure 9:**
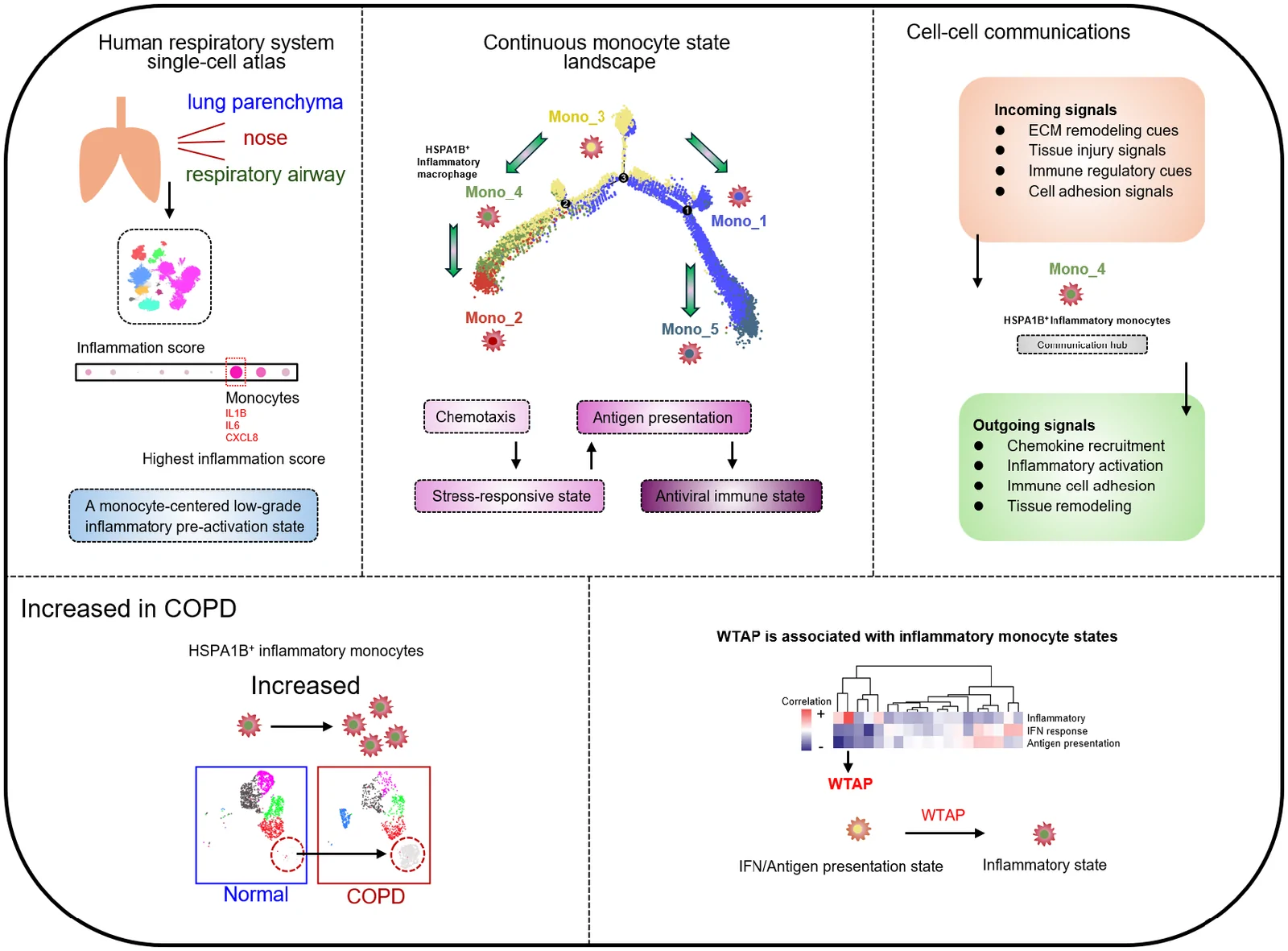
Schematic summary illustrating the association between WTAP and the HSPA1B^+^ inflammatory monocyte states in COPD. The schematic summarizes the association of WTAP with inflammatory monocyte features and related transcriptional programs in COPD.

## Discussion

Chronic inflammation represents a central pathological feature of COPD, yet the cellular states and regulatory mechanisms underlying persistent pulmonary immune activation remain incompletely understood. Although monocytes and macrophages have been extensively implicated in COPD pathogenesis, the functional organization of monocyte states within the human respiratory system remains poorly defined. In this study, by integrating single-cell transcriptomic datasets from multiple anatomical compartments of the healthy respiratory system and COPD lungs, we establish a high-resolution landscape of human respiratory monocyte heterogeneity and identify an HSPA1B^+^ inflammatory monocytes state characterized by enhanced inflammatory activity, cytokine/chemokine expression, and inflammatory transcriptional programs.

Traditionally, human monocytes have been classified into classical, intermediate, and non-classical subsets based on CD14 and CD16 expression patterns (Kapellos et al., 2019; Wong et al., 2012). However, increasing evidence indicates that monocyte identity extends beyond this binary classification and is shaped by tissue-specific environmental cues (Mulder et al., 2021; Teh et al., 2023). Our identification of HSPA1B^+^ inflammatory monocytes further supports this concept by demonstrating that monocytes within healthy respiratory tissues contain diverse functional states, including an inflammatory transcriptional program. Rather than representing a pathological cell population exclusively induced during disease, HSPA1B^+^ inflammatory monocytes were observed in healthy respiratory tissues and showed an inflammatory transcriptional profile. This finding extends current models of pulmonary immune regulation by suggesting that chronic inflammatory diseases may involve dysregulation of physiological immune programs. Similar concepts have emerged in other inflammatory contexts, where disease-associated immune states often reflect quantitative amplification or functional remodeling of existing cellular programs rather than emergence of entirely novel cell populations (Fabre et al., 2023; Yang Z. et al., 2026).

Single-cell trajectory analysis provides further insight into the transcriptional organization of monocyte functional states. Our data reveal a continuous transcriptional landscape in which different monocyte subsets occupy distinct positions along an inferred transcriptional ordering. Within this trajectory, Mono_3 cluster exhibits an earlier position with enhanced interferon-response signatures, consistent with a distinct transcriptional state. Along this continuum, HSPA1B^+^ inflammatory monocytes represent a state enriched for inflammatory signaling, cytokine production, and NF-κB-associated programs, whereas another branch is associated with antigen-presentation features. These findings suggest that monocyte functional specialization may be shaped by dynamic environmental signals rather than predetermined lineage commitment (Amorim et al., 2022; Ji et al., 2026). Importantly, while pseudotime analysis provides a framework for understanding transcriptional ordering and relationships among monocyte states, it does not establish definitive cellular lineage relationships. Therefore, HSPA1B^+^ inflammatory monocytes should be interpreted as occupying a distinct functional position within a continuous monocyte state landscape rather than representing an obligate differentiation endpoint. Future lineage-tracing and spatial approaches will be required to further define the developmental relationship among these monocyte states.

A major finding of our study is that COPD lungs exhibit selective enrichment of the HSPA1B^+^ inflammatory monocytes program. Comparative analysis between healthy and COPD tissues demonstrated that the transcriptional characteristics of COPD-associated inflammatory monocytes closely resemble those of HSPA1B^+^ monocytes identified in healthy respiratory tissues. These findings suggest that COPD-associated inflammation may involve altered representation and functional features of inflammatory monocyte states rather than the generation of completely distinct pathogenic monocyte populations. Chronic exposure to cigarette smoke, environmental pollutants, microbial components, and inflammatory mediators may provide persistent signals that favor expansion or maintenance of inflammatory monocyte state within diseased lung environments (Corleis et al., 2023; Gangwar et al., 2020; Oliveira da Silva et al., 2017). Interestingly, this inflammatory state was enriched in COPD lung tissues and bronchoalveolar lavage fluid but showed limited representation in peripheral blood monocytes from both healthy individuals and COPD patients. This observation suggests that tissue-associated environmental cues may play a dominant role in shaping monocyte functional states, highlighting the importance of studying immune cells within their native tissue context rather than solely relying on circulating immune populations.

Beyond transcriptional characterization, our cell-cell communication analysis reveals that HSPA1B^+^ inflammatory monocytes show prominent signaling activity within the inferred pulmonary stromal-immune-vascular communication network. These cells exhibit strong bidirectional signaling capacity, with inferred interactions involving multiple tissue compartments. MIF signaling emerged as one of the dominant communication pathways associated with monocyte regulation. In addition, extracellular matrix-associated signals, including COLLAGEN and LAMININ pathways, together with immune regulatory signals such as ANNEXIN and GALECTIN, suggest that HSPA1B^+^ inflammatory monocytes integrate structural and immune-derived environmental information to maintain their functional state. Meanwhile, CXCL and adhesion-associated outgoing signals indicate that these cells may participate in coordinating immune cell recruitment and vascular interactions. Thus, HSPA1B^+^ inflammatory monocytes may serve as immune sensing and communication nodes that connect tissue environmental changes with local inflammatory responses.

Epitranscriptomic regulation has emerged as an important mechanism controlling immune cell plasticity (Wang et al., 2021); however, how m^6^A regulatory networks shape human pulmonary monocyte states remains poorly understood. Here, we identify WTAP, a core component of the m^6^A methyltransferase complex, as a factor associated with the HSPA1B^+^ inflammatory monocytes program. WTAP expression positively correlates with inflammatory activity, whereas WTAP perturbation in inflammatory monocyte models reduces expression of inflammatory mediators while enhancing interferon-response and antigen-presentation programs. These findings suggest that WTAP-associated transcriptional regulation is linked to inflammatory monocyte functional states and the balance between inflammatory activation and immune regulatory programs. However, our data do not establish WTAP as a COPD-specific driver or demonstrate direct regulation of individual inflammatory transcripts. Moreover, because direct m6A modification was not measured in this study, these findings do not establish a direct WTAP–m6A regulatory mechanism. Instead, WTAP should be considered as a molecular factor associated with inflammatory monocyte remodeling. Future studies using cell-specific genetic models and transcriptome-wide m^6^A mapping will be required to define the direct molecular targets and mechanisms underlying WTAP-associated regulation.

Beyond identifying WTAP as a molecular factor associated with inflammatory monocyte activation, our findings raise the possibility that epitranscriptomic mechanisms could influence chronic pulmonary inflammation. Unlike broad immunosuppressive approaches that may compromise host defense, targeting WTAP-associated pathways could potentially alter inflammatory monocyte programs by reducing excessive cytokine production while preserving interferon-mediated immune surveillance and antigen-presentation capacity. However, given the essential roles of WTAP and m6A-related regulatory processes in fundamental cellular processes, the potential therapeutic implications of this pathway will require careful evaluation of cell-type specificity and context-dependent effects. Future studies using myeloid-specific WTAP perturbation models, selective m^6^A regulatory modulators, and COPD-relevant experimental systems will be necessary to determine whether WTAP inhibition can attenuate inflammatory remodeling while maintaining protective pulmonary immune functions.

Several limitations should be considered. First, although integrated single-cell analysis revealed conserved inflammatory monocyte state across healthy and COPD respiratory tissues, the cross-sectional nature of available datasets limits our ability to determine temporal relationships between HSPA1B^+^ monocyte remodeling and COPD progression. Longitudinal studies will be required to determine whether changes in this monocyte state correlate with disease severity, exacerbation frequency, or therapeutic response. Second, while trajectory analysis provides insights into the inferred transcriptional relationships among monocyte states, experimental lineage-tracing approaches are necessary to determine the developmental relationships among these populations. Third, although WTAP perturbation experiments support its functional contribution to inflammatory monocyte regulation, further studies are required to determine whether targeting WTAP-associated pathways can modulate pulmonary inflammation *in vivo*. Conditional genetic models and COPD-relevant experimental systems will be essential to evaluate the therapeutic potential of this regulatory axis. Finally, spatial transcriptomic and multi-omic approaches will be valuable for defining the localization, molecular interactions, and regulatory networks controlling HSPA1B^+^ inflammatory monocytes within the complex lung microenvironment.

Collectively, this study establishes a functional landscape of human respiratory monocyte states and identifies HSPA1B^+^ inflammatory monocytes as an inflammatory state associated with pulmonary inflammatory remodeling. Our findings suggest that COPD-related immune dysregulation involves increased representation and transcriptional modification of inflammatory monocyte programs. Furthermore, identification of WTAP as a factor associated with inflammatory monocyte transcriptional regulation provides new insights into the epitranscriptomic mechanisms governing monocyte inflammatory plasticity. These findings advance our understanding of pulmonary immune organization and provide a basis for further investigation of inflammatory monocyte states and their associated regulatory mechanisms in chronic respiratory diseases.

## Data Availability

The datasets presented in this study can be found in online repositories. The names of the repository/repositories and accession number(s) can be found in the article/supplementary material.
